# On *p*-adic *L*-functions for symplectic representations of $\text {GL}(N)$ over number fields

**DOI:** 10.1007/s40687-026-00635-w

**Published:** 2026-05-25

**Authors:** Chris Williams

**Affiliations:** School of Mathematical Sciences, University of Nottingham, Nottingham, NG7 2RD UK

**Keywords:** Primary 11F33, 11F67, Secondary 11R23

## Abstract

Let *F* be a number field and $\pi$ a regular algebraic cuspidal automorphic representation of $\textrm{GL}_N(\mathbb {A}_F)$ of symplectic type. When $\pi$ is spherical at all primes $\mathfrak {p}|p$, we construct a *p*-adic *L*-function attached to any regular non-critical spin *p*-refinement $\tilde{\pi }$ of $\pi$ to *Q*-parahoric level, where *Q* is the (*n*, *n*)-parabolic. More precisely, we construct a distribution $L_p(\tilde{\pi })$ on the Galois group $\textrm{Gal}_p$ of the maximal abelian extension of *F* unramified outside $p\infty$ and show that it interpolates all the standard critical *L*-values of $\pi$ at *p* (including, for example, cyclotomic and anticyclotomic variation when *F* is imaginary quadratic). We show that $L_p(\tilde{\pi })$ satisfies a natural growth condition; in particular, when $\tilde{\pi }$ is ordinary, $L_p(\tilde{\pi })$ is a (bounded) measure on $\textrm{Gal}_p$. As a corollary, when $\pi$ is unitary, has very regular weight, and is *Q*-ordinary at all $\mathfrak {p}|p$, we deduce non-vanishing $L(\pi \times (\chi \circ N_{F/\mathbb {Q}}),1/2) \ne 0$ of the twisted central value for all but finitely many Dirichlet characters $\chi$ of *p*-power conductor.

## Introduction

The special values of *L*-functions are highly important in modern number theory and are the subject of a vast network of conjectures, including:

(I) The *Beilinson and Bloch–Kato conjectures*, which describe arithmetic data in terms of the complex analytic properties of special values. As a special case, non-vanishing of a special value should force finiteness of an associated Selmer group.
(II) *Deligne’s conjecture* [21], which predicts that the special values, ostensibly arbitrary transcendental numbers, are algebraic after scaling by controlled complex ‘periods’.
(III) The *Coates–Perrin–Riou/Panchishkin conjectures* [19, 20, 36] that predict these special *L*-values satisfy deep *p*-adic congruences, generalising Kummer’s congruences for the Riemann zeta function.

The congruences predicted by (III) are encoded in the existence of a *p-adic L-function*. Beautiful objects in their own right, *p*-adic *L*-functions are fundamental in Iwasawa theory and have led to substantial results towards (I), including special cases of the Birch–Swinnerton–Dyer conjecture.

A very general setting to consider these questions is that of regular algebraic cuspidal automorphic representations (*RACARs*) $\pi$ of ${{\,\textrm{GL}\,}}_N$ over a number field *F*. In this paper, we attack (III) by constructing *p*-adic *L*-functions for those $\pi$ which are *symplectic* (*RASCARs*, for RA-symplectic-CARs).

### Main result

Let us state our main result more precisely. Let *F* be an arbitrary number field, $G :=\textrm{Res}_{F/\textbf{Q}}({{\,\textrm{GL}\,}}_N)$, and $\pi$ a RASCAR of $G(\textbf{A})$. This forces $N = 2n$ to be even, and $\pi \cong \pi ^\vee \otimes \eta$ to be essentially self-dual, for a Hecke character $\eta$ of $F^\times \backslash \textbf{A}_F^\times$. Further, $\pi$ is symplectic if and only if it admits a Shalika model, if and only if it is a functorial transfer from $\textrm{Res}_{F/\textbf{Q}}\textrm{GSpin}_{2n+1}$ [1, 2, 24]. In this setting, the automorphic realisation of Deligne’s conjecture was recently proved—including the period relations at infinity—in work of Jiang–Sun–Tian [31].

Let *p* be a prime and assume that $\pi$ is spherical at all primes $\mathfrak {p}$ of *F* above *p*. Then $\pi _{\mathfrak {p}} = {{\,\textrm{Ind}\,}}_B^G \theta _{\mathfrak {p}}$ is an unramified principal series, the normalised induction of some unramified character $\theta _{\mathfrak {p}} = (\theta _{\mathfrak {p},1},...,\theta _{\mathfrak {p},2n})$ (where *B* is the upper-triangular Borel). Let $Q \subset G$ be the standard parabolic subgroup with Levi $H :=\textrm{Res}_{F/\textbf{Q}}({{\,\textrm{GL}\,}}_n\times {{\,\textrm{GL}\,}}_n)$, and let $J_{\mathfrak {p}} \subset {{\,\textrm{GL}\,}}_{2n}(F_{\mathfrak {p}})$ be the parahoric subgroup of type *Q*. If $\mathfrak {p}|p$, a regular *Q*-refinement to level $J_{\mathfrak {p}}$ is a choice of simple Hecke eigenvalue $\alpha _{\mathfrak {p}}$ on $\pi _{\mathfrak {p}}^{J_{\mathfrak {p}}}$, defined precisely in Definition 3.3. We assume this choice is *spin* (Definition 3.4), in that it interacts well with the Shalika model; as explained in the introduction of [11], we expect this is essential for constructions of *p*-adic *L*-functions via Shalika models. Write $\tilde{\pi }$ for $\pi$ with such a choice of regular spin *Q*-refinement at each $\mathfrak {p}|p$. We assume $\tilde{\pi }$ to be non-*Q*-critical (Definition 7.1). This is satisfied if the integrally normalised eigenvalues $\alpha _{\mathfrak {p}}^\circ$, defined in §7.1, have non-*Q*-critical slope. In particular, if $\tilde{\pi }$ is ordinary (i.e. each $v_p(\alpha _{\mathfrak {p}}^\circ ) = 0$), then it is non-*Q*-critical.

We say a Hecke character $\chi : F^\times \backslash \textbf{A}_F^\times \rightarrow \textbf{C}^\times$ is *critical* for $\pi$ if $s=1/2$ is a Deligne-critical value of $L(\pi \times \chi ,s)$, and then, we define $L(\pi ,\chi ) :=L(\pi \times \chi ,1/2)$. The critical characters are determined by their infinity type and the weight $\lambda$ of $\pi$. For general *F*, this *L*-function can have many different critical regions; see e.g. the pictures in [33, p.1605] or [16, §4.1]. We focus here on the ‘standard’ critical range^1^, corresponding to the ‘balanced weight’ condition in [31] (and, when *F* is imaginary quadratic, region $\Sigma ^{(1)}$ in [33]). We describe this range in Lemma 2.4. For example, when *F* is imaginary quadratic, this gives a square of standard critical infinity types (see §7.4). Crucially, these standard critical values admit a representation-theoretic interpretation via branching laws for $H \subset G$, described in Lemma 2.6.

We write $\textrm{Crit}_p(\pi )$ for the set of standard critical characters that have *p*-power conductor. Let ${{\,\textrm{Gal}\,}}_p$ be the Galois group of the maximal abelian extension of *F* unramified outside $p\infty$. Attached to any $\chi \in \textrm{Crit}_p(\pi )$ is a canonical character $\chi _{[p]}$ on ${{\,\textrm{Gal}\,}}_p$ (see §2.5).

We construct a *p*-adic *L*-function attached to $\tilde{\pi }$, in the following sense:

#### Theorem A

For any choice of isomorphism , there exist a finite extension $L/\textbf{Q}_p$ and an *L*-valued locally analytic distribution $L_p^{i_p}(\tilde{\pi })$ on ${{\,\textrm{Gal}\,}}_p$ such that:

- For any $\chi \in \textrm{Crit}_p(\pi )$ of conductor $\prod _{\mathfrak {p}|p} \mathfrak {p}^{\beta _{\mathfrak {p}}}$, we have
  $$\begin{aligned} L_p^{i_p}(\tilde{\pi }, \chi )&:=i_p^{-1} \left( \int _{{{\,\textrm{Gal}\,}}_p} \chi _{[p]} \cdot \textrm{d}L_p^{i_p}(\tilde{\pi })\right) \\&= A \cdot \tau (\chi _f)^n \cdot \prod _{\mathfrak {p}|p} e(\pi _{\mathfrak {p}},\chi _{\mathfrak {p}}) \cdot \frac{L^{(p)}(\pi ,\chi )}{\Omega _{\pi ,\chi _\infty }}, \end{aligned}$$
  where *A* is a constant defined in (5.15), $\Omega _{\pi ,\chi _\infty } \in \textbf{C}^\times$ is defined in Definition 3.9, and
  $$\begin{aligned} e(\pi _{\mathfrak {p}},\chi _{\mathfrak {p}})=\left\{ \begin{array}{cl} q_{\mathfrak {p}}^{\beta _{\mathfrak {p}}\left( \tfrac{n^2-n}{2}\right) } \alpha _{\mathfrak {p}}^{-\beta _{\mathfrak {p}}} & : \chi _{\mathfrak {p}} \text { ramified},\\ \displaystyle \prod _{i=n+1}^{2n} \frac{1-\theta _{\mathfrak {p},i}^{-1}\chi _{\mathfrak {p}}^{-1}(\varpi _{\mathfrak {p}})q_{\mathfrak {p}}^{-1/2}}{1-\theta _{\mathfrak {p},i}\chi _{\mathfrak {p}}(\varpi _{\mathfrak {p}})q_{\mathfrak {p}}^{-1/2}} & : \chi _{\mathfrak {p}} \text { unramified}.\end{array}\right. \end{aligned}$$
- $L_p^{i_p}(\tilde{\pi })$ is admissible of growth $(h_{\mathfrak {p}})_{\mathfrak {p}|p}$, where $h_{\mathfrak {p}} :=v_{p}(\alpha _{\mathfrak {p}}^\circ )$.

The growth condition is described in Definition 7.4. In particular, if $\tilde{\pi }$ is ordinary, then $L_p^{i_p}(\tilde{\pi })$ is a *bounded* distribution, that is, a *p*-adic measure. Here $L^{(p)}$ is the *L*-function with the Euler factors at *p* removed, $\tau (\chi _f)$ is a Gauss sum, $q_{\mathfrak {p}} = N_{F/\textbf{Q}}(\mathfrak {p})$, and $\varpi _{\mathfrak {p}}$ is a uniformiser. This agrees exactly with the conjectures of Coates–Perrin-Riou/Panchishkin [36, Conj. 6.2], except possibly the term $\Omega _{\pi ,\chi _\infty }$, which we comment on in Remark 3.11.

If *F* is imaginary quadratic, and $\tilde{\pi }$ has non-*Q*-critical slope, then (a) and (b) determine $L_p^{i_p}(\tilde{\pi })$ uniquely by [35]. Analogous unicity results for *F* totally real are [12, Prop. 6.25].

To our knowledge, Theorem A gives the first such construction beyond the special cases of ${{\,\textrm{GL}\,}}_2$ (over any *F*) or *F* totally real; see §1.3 for a summary of previous results. The totally real case was handled in [12, 23]. The construction we give is inspired by those works, particularly the overconvergent approach of [12]. There are some additional features in the general number field setting, which we briefly summarise.

1. Notably, whilst Leopoldt’s conjecture predicts that $L_p(\tilde{\pi })$ is 1-variabled when *F* is totally real, in general our *p*-adic *L*-functions can be many-variabled. For example, when $F = \textbf{Q}$ we have ${{\,\textrm{Gal}\,}}_p\cong \textbf{Z}_p^\times$, and one only has cyclotomic variation; when *F* is imaginary quadratic, one has a two-variable *p*-adic *L*-function, with both cyclotomic and anticyclotomic variation.
2. The method uses automorphic cycles/modular symbols. The totally real (resp. ${{\,\textrm{GL}\,}}_2$) constructions relied on a certain numerical coincidence that these cycles have dimension equal to the top degree (resp. bottom degree) in which cuspidal cohomology for *G* contributes. This rendered some relevant cohomology groups 1-dimensional, making certain choices unique up to scalar. In the general number field case, we work in the middle of the cuspidal range, where the analogous groups are never 1-dimensional.

We handle (1) by blending the methods of [12] with earlier work [7, 42] of the author and Barrera for ${{\,\textrm{GL}\,}}_2$. Problem (2) is more serious, and for this we exploit works of Lin–Tian [34] and Jiang–Sun–Tian [31]. They nailed down good elements in these higher-dimensional cohomology groups and proved not only the non-vanishing hypothesis of attached zeta integrals at infinity, but also the expected period relations at infinity, at least in the cyclotomic direction (see Remark 3.11). We hope that the period relations in *all* directions can be extracted via similar methods, but do not address that here, instead focusing on the *p*-adic interpolation.

We imagine the following special case might be of particular interest. Let $\mathcal {F}$ be a cohomological Siegel modular form on $\textrm{GSp}_4/\textbf{Q}$ and base-change it to an imaginary quadratic field *F*. Under appropriate assumptions, we may apply our construction to the Langlands transfer of $\mathcal {F}/F$ to ${{\,\textrm{GL}\,}}_4/F$ (via [1]), giving a two-variable *p*-adic spin *L*-function for $\mathcal {F}/F$, including anticyclotomic variation. For ${{\,\textrm{GL}\,}}_2$, such constructions for base-change modular forms have had important arithmetic consequences, such as proofs of one divisibility of the Iwasawa Main Conjecture [40].

### Application: non-vanishing of central twists

When $\tilde{\pi }$ is ordinary at each $\mathfrak {p}|p$, then as mentioned above, our construction yields a *p*-adic measure. As an immediate application, we get the following generalisation of a result of Dimitrov–Januszewski–Raghuram (who in [23] treated the case *F* totally real). Let $\lambda = (\lambda _\sigma )_{\sigma \in \Sigma }$ denote the weight of $\pi$, where $\lambda _\sigma = (\lambda _{\sigma ,1},...,\lambda _{\sigma ,2n}) \in \textbf{Z}^{2n}$ is dominant and $\Sigma$ is the set of embeddings $\sigma : F \hookrightarrow \textbf{C}$ (see §2.3).

#### Theorem B

Let $\pi$ be a unitary RASCAR of $G(\textbf{A})$, and suppose

1.1 $$\begin{aligned} \lambda _{\sigma ,n} > \lambda _{\sigma ,n+1} \qquad \text { for all }\sigma \in \Sigma . \end{aligned}$$

Suppose there exists a rational prime *p* such that $\pi _{\mathfrak {p}}$ is spherical and *Q*-ordinary for all $\mathfrak {p}|p$. Then for all but finitely many Dirichlet characters $\chi$ of *p*-power conductor, we have non-vanishing of the twisted central *L*-value

$$\begin{aligned} L(\pi \times (\chi \circ N_{F/\mathbb {Q}}),\tfrac{1}{2}) \ne 0. \end{aligned}$$

Here *Q-ordinarity* is in the sense of [28, §1.1]. The unitary assumption ensures $s=1/2$ is a Deligne-critical value of $L(\pi ,s)$.

Given Theorem A, the proof of Theorem B is simple. The argument is literally identical to [23, §4.4], so we give only a sketch, and refer the reader there for full details.

#### Proof

Since $\pi _{\mathfrak {p}}$ is *Q*-ordinary for all $\mathfrak {p}|p$, by [23, Lem. 4.4] there is a (unique) regular spin ordinary *Q*-refinement $\tilde{\pi }$ of $\pi$. Let $L_p^{i_p}(\tilde{\pi })$ the *p*-adic *L*-function from Theorem A. By [29] (see [12, Lem. 7.4]), the weight condition forces existence of some $j \ne 0$ such that the (non-central) twisted *L*-values $L(\pi \times (\chi \circ N_{F/\textbf{Q}}),j+1/2)$ are nonzero for all Dirichlet characters $\chi$.

If $\chi$ is a Dirichlet character of *p*-power conductor, then $\chi \circ N_{F/\textbf{Q}}$ is a cyclotomic character of ${{\,\textrm{Gal}\,}}_p$. The restriction of $L_p^{i_p}(\tilde{\pi })$ to the cyclotomic line is a bounded rigid analytic function on the disjoint union of a finite number of open unit discs. By the interpolation property and non-vanishing at non-central values, the *p*-adic *L*-function is nonzero on each disc that contains characters of the form $\chi \circ N_{F/\textbf{Q}}$. By Weierstrass preparation, it has finitely many zeros in each such disc, hence on the cyclotomic line; so the theorem follows from the interpolation property. $\square$

If one drops assumption (1.1), the theorem instead becomes: if there exists a $\chi$ as in the theorem such that $L(\pi \times (\chi \circ N_{F/\mathbb {Q}}),1/2) \ne 0$, then there are infinitely many such $\chi$.

In the ${{\,\textrm{GL}\,}}_4$ case, after transferring via [1], this gives non-vanishing of many twisted central values of spin *L*-functions of (very regular weight, cohomological, Klingen-ordinary) genus 2 Siegel modular forms over number fields.

### Relation to the literature

This work generalises (and is visibly inspired by) many other constructions. We summarise a few. In the case of ${{\,\textrm{GL}\,}}(2)$, i.e. $n=1$, every RACAR is a RASCAR, and our main results/methods specialise exactly to those of [7], and the results/methods of that paper in turn followed the earlier works [6, 37, 42] (over $\textbf{Q}$, totally real, and imaginary quadratic base fields respectively). These methods were later used in [5, 8, 9, 14, 15] to vary *p*-adic *L*-functions in families.

In the case of general ${{\,\textrm{GL}\,}}(2n)$, when *F* is totally real, our main result specialises exactly to [12, Thm. 6.23]. Earlier constructions in the ordinary situation were given in [4, 23, 26]. This construction was then used in [12, 13] to construct and study *p*-adic families, and—under some technical hypotheses—to vary *p*-adic *L*-functions over these families.

The variation in the present construction in families would be very interesting. The methods of these earlier papers—which worked in either top or bottom cohomological degree—do not apply. Fundamental difficulties include: controlling the families; constructing classes in the correct cohomological degree; and showing that these classes interpolate the ‘good’ elements from [31].

Rohrlich [39] proved a ${{\,\textrm{GL}\,}}_2$ analogue of Theorem B. The case *F* totally real is [23]. For ${{\,\textrm{GL}\,}}_4/\textbf{Q}$, an analogous result for all weights and unitary CARs was recently proved in [38].

Finally, in Theorems A and B it should be possible to weaken the assumption that $\pi _{\mathfrak {p}}$ is spherical at all $\mathfrak {p}|p$ to *Q*-parahoric-spherical using forthcoming work of Dimitrov–Jorza [22].

## Preliminaries

### Notation

Let *F* be a number field of degree $d = r+2s$, where *F* has *r* real embeddings and *s* pairs of complex embeddings. Let $\Sigma :=\{\sigma : F \hookrightarrow \textbf{C}\} = \Sigma _{\textbf{R}} \sqcup \Sigma _{\textbf{C}}$ be the union of the real and complex embeddings. Each $\sigma \in \Sigma$ has a conjguate $c\sigma$, and $\sigma = c\sigma$ if and only if $\sigma (F) \subset \textbf{R}$. Fix a rational prime *p* and an isomorphism . Let $F^{p\infty }$ be the maximal abelian extension of *F* unramified outside $p\infty$, and let ${{\,\textrm{Gal}\,}}_p:=\textrm{Gal}(F^{p\infty }/ F)$ be its Galois group. Let $\psi$ be the standard non-trivial additive character of $F\backslash \textbf{A}_F$ (as e.g. fixed in [23, §4.1]).

If *v* is a place of *F*, we write $F_v$ for the completion of *F* at *v*, $\mathcal {O}_v$ for its ring of integers, and $\textbf{F}_v$ for its residue field. We fix a choice of uniformiser $\varpi _v \in \mathcal {O}_v$.

Let $G = \textrm{Res}_{F/\textbf{Q}}{{\,\textrm{GL}\,}}_{2n}$, $B = TN$ be the upper-triangular Borel sugroup in *G*, *T* the diagonal torus, and *N* the unipotent. Let $H = \textrm{Res}_{F/\textbf{Q}}({{\,\textrm{GL}\,}}_n\times {{\,\textrm{GL}\,}}_n)$, with $\iota : H \hookrightarrow G$, $$(h_1,h_2) \mapsto \left( {\begin{smallmatrix}h_1 &  \\  &  h_2\end{smallmatrix}}\right) $$. We may abuse notation and write, e.g. $B(F_{v})$ for the upper-triangular matrices in ${{\,\textrm{GL}\,}}_{2n}(F_v)$.

Let $K_\infty =C_\infty Z_\infty \subset G(\textbf{R})$, where $Z_\infty$ is the centre and $C_\infty = \textrm{O}_{2n}(\textbf{R})^r \times \textrm{U}_{2n}(\textbf{C})^s$ is the maximal compact subgroup of $G(\textbf{R})$. If *A* is a reductive real Lie group, then $A^\circ$ denotes the connected component of the identity.

Let $Q = HN_Q$ be the standard parabolic with Levi *H*. For a prime $\mathfrak {p}|p$ of *G*, let $J_{\mathfrak {p}} :=\{g \in {{\,\textrm{GL}\,}}_{2n}(\mathcal {O}_{\mathfrak {p}}) : g \hspace{2pt}(\textrm{mod}\hspace{2pt}\mathfrak {p}) \in Q(\textbf{F}_{\mathfrak {p}})\} \subset {{\,\textrm{GL}\,}}_{2n}(F_{\mathfrak {p}})$ be the parahoric subgroup of type *Q*, and $J_p :=\prod _{\mathfrak {p}|p}J_{\mathfrak {p}} \subset G(\textbf{Q}_p)$.

### RASCARs

Let $\pi$ be a regular algebraic cuspidal automorphic representation (RACAR) of $G(\textbf{A})$. If $\pi$ is essentially self-dual, i.e. there exists some Hecke character $\eta$ such that $\pi ^\vee \cong \pi \otimes \eta ^{-1}$, then $L(\pi \times \pi ^\vee ,s) = L(\pi , \textrm{Sym}^2\otimes \eta ^{-1},s)L(\pi ,\wedge ^2\otimes \eta ^{-1}, s)$. This has a simple pole at $s=1$, which occurs either in the symmetric or exterior square *L*-function. Then:

#### Definition 2.1

We say that $\pi$ has *symplectic type* and call it a *RASCAR* (RA-symplectic-CAR), if any (hence all) of the following equivalent conditions hold:

1. The exterior square *L*-function $L(\pi ,\wedge ^2 \otimes \eta ^{-1},s)$ has a pole at $s=1$;
2. $\pi$ is the functorial transfer of an irreducible generic cuspidal automorphic representation $\Pi$ of $\textrm{GSpin}_{2n+1}(\textbf{A}_F)$ with central character $\eta$;
3. $\pi$ admits a non-trivial $(\eta ,\psi )$-Shalika model.

Recall $\pi$ has a $(\eta ,\psi )$-Shalika model if there is an intertwining $\mathcal {S}_{\psi }^\eta : \pi \hookrightarrow {{\,\textrm{Ind}\,}}_{\mathcal {S}(\textbf{A})}^{G(\textbf{A})} (\eta \otimes \psi )$, where $$\mathcal {S}= \big \{s(h,X) = \left( {\begin{smallmatrix}h &  \\  &  h\end{smallmatrix}}\right) \left( {\begin{smallmatrix}1_n &  X\\  &  1_n\end{smallmatrix}}\right) : h \in {{\,\textrm{GL}\,}}_n, X \in \textrm{M}_n\big \}$$ is the Shalika group and $(\eta \otimes \psi )(s(h,X)) = \eta (\det (h))\cdot \psi (\textrm{Tr}(X))$. Our conventions are summarised in [12, §2.6].

In the definition, (1)$\iff$(3) was substantially proved in [30], and (1)$\iff$(2) in [1, 2]. For the equivalence in the exact form above, see [25, Thm. 5.1].

### Weights

Let $X^*(T)$ be the set of algebraic weights for *T*. A general $\lambda \in X^*(T)$ has form $\lambda = (\lambda _\sigma )_{\sigma \in \Sigma }$, where $\lambda _\sigma = (\lambda _{\sigma ,1}, ..., \lambda _{\sigma ,2n}) \in \textbf{Z}^{2n}$. Let $X_+^*(T)$ be the set of *dominant* weights, where $\lambda _{\sigma ,i} \geqslant \lambda _{\sigma ,i+1}$ for all $\sigma$ and *i*. Attached to any $\lambda \in X_+^*(T)$ is an algebraic representation $V_\lambda$ of *G* of highest weight $\lambda$, with dual $V_\lambda ^\vee$. We can decompose $V_\lambda = \otimes _{\sigma \in \Sigma } V_{\lambda ,\sigma }$, $V_{\lambda }^\vee = \otimes _{\sigma \in \Sigma }V_{\lambda ,\sigma }^\vee$, where $V_{\lambda ,\sigma }$ is the algebraic ${{\,\textrm{GL}\,}}_{2n}$-representation of highest weight $\lambda _{\sigma }$, with *G* acting via $\sigma$.

#### Definition 2.2

We say $\lambda \in X_+^*(T)$ is *pure* if there exists $\textsf{w}\in \textbf{Z}$ such that

$$\begin{aligned} \lambda _{\sigma ,i} + \lambda _{c\sigma ,2n+1-i} = \textsf{w}\qquad \text {for all }\sigma \text { and }i. \end{aligned}$$

We write $X_0^*(T) \subset X_+^*(T)$ for the space of pure dominant weights.

Let $\pi$ be RASCAR of $G(\textbf{A})$. Let $\mathfrak {g}_\infty :=\textrm{Lie}(G(\textbf{R}))$. As in [18], there is a unique $\lambda \in X^*_0(T)$, the *weight* of $\pi$, such that

$$\begin{aligned} \textrm{H}^\bullet (\mathfrak {g}_\infty ,K_\infty ^\circ ; \pi _\infty \otimes V_\lambda ^\vee (\textbf{C})) \ne 0. \end{aligned}$$

Here $\lambda$ is pure by [18, Lem. 4.9]. Since $\pi$ is a RASCAR, $\lambda$ is further restricted: by [34, Thm. 2.1], for all $\sigma \in \Sigma$ there exists $\textsf{w}_\sigma \in \textbf{Z}$ such that $\lambda _{\sigma ,i} + \lambda _{\sigma ,2n+1-i} = \textsf{w}_\sigma$ (i.e. each $\lambda _\sigma$ is *individually* pure; see also [31, Prop. 2.17] for the statement in this form). We have $\textsf{w}_\sigma = \textsf{w}$ for $\sigma \in \Sigma _{\textbf{R}}$, and $\textsf{w}_\sigma + \textsf{w}_{c\sigma } = 2\textsf{w}$ for $\sigma \in \Sigma _{\textbf{C}}$. We let $\underline{\textsf{w}} = (\textsf{w}_\sigma )_{\sigma \in \Sigma } \in \textbf{Z}^{\Sigma }$.

### Critical *L*-values

Let $\pi$ be a RASCAR with standard *L*-function $L(\pi ,s)$. For a Hecke character $\chi : F^\times \backslash \textbf{A}_F^\times \rightarrow \textbf{C}^\times$, let $\pi \times \chi$ denote the RASCAR with $G(\textbf{A})$-action twisted by $\chi$. We write $L(\pi ,\chi ) :=L(\pi \times \chi ,1/2)$.

#### Definition 2.3

Let $\chi$ be an algebraic Hecke character with infinity type $\textbf{j} = (j_\sigma )_{\sigma \in \Sigma } \in \textbf{Z}^\Sigma$, and let $\pi$ have weight $\lambda$. We say $\chi$ is a *standard critical value for* $L(\pi ,-)$ if

2.1 $$\begin{aligned} -\lambda _{\sigma ,n} \leqslant j_\sigma \leqslant -\lambda _{\sigma ,n+1} \qquad \forall \sigma \in \Sigma . \end{aligned}$$

In this case, we say $\chi$ is *critical for* $\lambda$. We write $\textrm{Crit}(\pi )$ for the set of all such $\chi$.

#### Lemma 2.4

If $\chi \in \textrm{Crit}(\pi )$, then $s=1/2$ is a Deligne-critical value of $L(\pi \times \chi ,s)$.

#### Proof

The RACAR $\pi \times \chi$ has weight $\lambda + \textbf{j} = (\lambda _\sigma + (j_\sigma ,...,j_\sigma ))_\sigma$. By [31, Prop. 2.20], $s=1/2$ is a standard critical value of $L(\pi \times \chi ,s)$ if and only if $-(\lambda _{\sigma ,n} + j_\sigma ) \leqslant 0 \leqslant -(\lambda _{\sigma ,n+1} + j_\sigma )$ for all $\sigma \in \Sigma$. This is equivalent to (2.1). $\square$

#### Remark 2.5

For $\textbf{j}$ in this range, $\lambda +\textbf{j}$ is a balanced weight in the sense of [31]. There are other Deligne-critical values, but for these $\lambda +\textbf{j}$ is not balanced.

Crucially $\textrm{Crit}(\pi )$ also admits a representation-theoretic description. For $\textbf{j}_1, \textbf{j}_2 \in \textbf{Z}^{\Sigma }$, let $V_{\textbf{j}_1,\textbf{j}_2}^H$ be the *H*-representation of highest weight $(\textbf{j}_1,\textbf{j}_2)$, that is, $\det _1^{\textbf{j}_1}\cdot \det _2^{\textbf{j}_2}$. The following is proved in the same way as [27, Prop. 6.3.1] and [31, Prop. 2.20], via [32, Thm. 2.1].

#### Lemma 2.6

A Hecke character $\chi$ of infinity type $\textbf{j} \in \textbf{Z}^\Sigma$ is in $\textrm{Crit}(\pi )$ if and only if

$$\begin{aligned} \textrm{Hom}_H(V_\lambda ^\vee , V^H_{\textbf{j},-\underline{\textsf{w}}-\textbf{j}}) \ne 0. \end{aligned}$$

In this case, $\textrm{Hom}_H(V_\lambda ^\vee , V^H_{\textbf{j},-\underline{\textsf{w}}-\textbf{j}})$ is a line, generated by some $\kappa _{\textbf{j}}$.

We will interpolate the standard critical *L*-values ‘at *p*’. As such, let

2.2 $$\begin{aligned} \textrm{Crit}_p(\pi ):=\{\chi \in \textrm{Crit}(\pi ) : \textrm{cond}(\chi ) = p^{\beta _0}\}, \end{aligned}$$

where $p^{\beta _0} :=\prod _{\mathfrak {p}|p}\mathfrak {p}^{\beta _{0,\mathfrak {p}}}$, for $\beta _0 = (\beta _{0,\mathfrak {p}}) \in \textbf{Z}_{\geqslant 0}^{\{\mathfrak {p}|p\}}$ a multiexponent.

### Hecke characters on ray class and Galois groups

Let $\chi$ be a Hecke character with infinity type $\textbf{j}\in \textbf{Z}[\Sigma ]$. We have an algebraic homomorphism $w^{\textbf{j}}: F^\times \longrightarrow \overline{\textbf{Q}}^\times$ given by $w^{\textbf{j}}(x) = \prod _{\sigma \in \Sigma }\sigma (x)^{j_\sigma }.$ This then induces maps

2.3 $$\begin{aligned} w^{\textbf{j}}_\infty :(F\otimes _{\textbf{Q}}\textbf{R})^\times \rightarrow \textbf{C}^\times \xrightarrow {i_p}\overline{\textbf{Q}}_p^\times , \qquad w^{\textbf{j}}_p: (F\otimes _{\textbf{Q}}\textbf{Q}_p)^\times \rightarrow \overline{\textbf{Q}}_p^\times . \end{aligned}$$

#### Definition 2.7

We define $\chi _{[p]}$ to be the function

$$\begin{aligned} \chi _{[p]}: \textbf{A}_F^\times \longrightarrow \overline{\textbf{Q}}_p^\times , \qquad x&\longmapsto \chi (x)\cdot \big [w_\infty ^{\textbf{j}}(x_\infty )\big ]^{-1} \cdot w_p^{\textbf{j}}(x_p) = \chi _\infty (\textrm{sgn}(x_\infty ))\cdot \chi _f(x) \cdot w_p^{\textbf{j}}(x_p), \end{aligned}$$

where $\textrm{sgn}(x_\infty ) \in \{\pm 1\}^{\Sigma _{\textbf{R}}}$. Via [41], this takes values in some finite extension $L/\textbf{Q}_p$. If $\mathfrak {f}$ is the conductor of $\chi$, then (as in e.g. [7, Prop. 2.4]) $\chi _{[p]}$ is a locally analytic character on the *p*-adic analytic group

$$\begin{aligned} \textrm{Cl}_F^+(\mathfrak {f}p^\infty ) :=F^\times \backslash \textbf{A}_F^\times /U(\mathfrak {f}p^\infty )F_\infty ^+, \end{aligned}$$

where $U(\mathfrak {f}p^\infty )$ is the group of elements of $\widehat{\mathcal {O}}_F^\times$ that are congruent to $1\hspace{2pt}(\textrm{mod}\hspace{2pt}\mathfrak {f}p^\infty )$.

Recall ${{\,\textrm{Gal}\,}}_p$ is the Galois group for the maximal abelian extension of *F* unramified outside $p\infty$. The structure of this group is described in detail in [12, §6.1]; in particular, the Artin reciprocity map induces an isomorphism , and we have an exact sequence

2.4 $$\begin{aligned} 0 \rightarrow \overline{\mathcal {O}}_{F,+}^\times \rightarrow (\mathcal {O}_F\otimes \textbf{Z}_p)^\times \rightarrow {{\,\textrm{Gal}\,}}_p\rightarrow \textrm{Cl}_F^+ \rightarrow 0. \end{aligned}$$

Via reciprocity, we may consider $\chi _{[p]}$ as a character of ${{\,\textrm{Gal}\,}}_p$.

### Locally symmetric spaces and local systems

If $K \subset G(\textbf{A}_f)$ is open compact, the locally symmetric space of level *K* is

$$\begin{aligned} S_K :=G(\textbf{Q})\backslash G(\textbf{A})/KK_\infty ^\circ . \end{aligned}$$

If *M* is a left $G(\textbf{Q})$-module, then we have an attached ‘archimedean’ local system $\mathcal {M}$ on $S_K$ given by the locally constant sections of

2.5 $$\begin{aligned} G(\textbf{Q})\backslash [G(\textbf{A})\times M]/KK_\infty ^\circ \longrightarrow S_K, \end{aligned}$$

where $\gamma (g,m)kz = (\gamma gkz, \gamma \cdot m)$ for $\gamma \in G(\textbf{Q}), g\in G(\textbf{A}), k\in K$, $z \in K_\infty ^\circ$ and $m \in M$. Similarly, if *M* is a left *K*-module, then we have an attached ‘*p*-adic’ local system on $S_K$, given by the local constant sections of (2.5), but with action $\gamma (g,m)kz = (\gamma gkz, k^{-1}\cdot m)$.

If *M* is a left $G(\textbf{Q}_p)$-module, then $G(\textbf{Q})$ acts via $G(\textbf{Q})\subset G(\textbf{Q}_p)$, and *K* acts via projection to $G(\textbf{Q}_p)$. We get two attached local systems $\mathcal {M}$ and $\mathscr {M}$, and these are isomorphic via the map $(g,m) \mapsto (g_p^{-1}\cdot m)$ of local systems. For more detail see [12, §2.3].

### Algebraic and analytic coefficient modules

#### Algebraic coefficients

If $\lambda \in X^\bullet (T)$ is a dominant algebraic weight, recall $V_\lambda$ is the algebraic *G*-representation of highest weight $\lambda$. If $\textbf{Q}_p\subset L \subset \overline{\textbf{Q}}_p$, we describe $V_\lambda (L)$ via algebraic induction $\textrm{Ind}_{\overline{B}}^{G}\lambda$; namely,

$$\begin{aligned} V_\lambda (L) :=\{f :G(\textbf{Q}_p) \rightarrow L : f\text { is algebraic}, f(\overline{b}g) = \lambda (\overline{b})f(g)\ \ \forall \overline{b} \in \overline{B}(\textbf{Q}_p)\}, \end{aligned}$$

where $\overline{B}$ is the opposite Borel. This space carries a natural left action of $G(\textbf{Q}_p)$ given by

2.6 $$\begin{aligned} (g\cdot f)(h) :=f(hg), \qquad g,h \in G(\textbf{Q}_p), \end{aligned}$$

inducing a dual left action on $V_\lambda ^\vee (L)$ by $g \cdot \mu (f) :=\mu (g^{-1}\cdot f)$. By §2.6, we have attached local systems $\mathcal {V}_\lambda ^\vee (L)$ and $\mathscr {V}_\lambda ^\vee (L)$, and a natural isomorphism .

For our *p*-adic interpolation, the subgroup $H \subset G$ plays a crucial role; its importance is that *G*/*H* is a spherical variety. It is thus convenient to consider $V_\lambda$ as a double algebraic induction, namely

2.7 $$\begin{aligned} V_\lambda \cong {{\,\textrm{Ind}\,}}_{\overline{Q}}^{G} {{\,\textrm{Ind}\,}}_{\overline{B}\cap H}^{H} \lambda \end{aligned}$$

(see [12, Lem. 3.6]). Here $\overline{Q}$ is the opposite parabolic to *Q* (that is, the (*n*, *n*)-block lower-triangular subgroup). Write $V_\lambda ^H = {{\,\textrm{Ind}\,}}_{\overline{B}\cap H}^{H} \lambda$ for the algebraic *H*-representation of highest weight $\lambda$, and denote the standard action of $h \in H$ on $v \in V_{\lambda }^H$ by $\langle h\rangle _\lambda \cdot v$. Then precisely, (2.7) means we can identify $V_\lambda (L)$ with the space of algebraic $f : G(\textbf{Q}_p) \rightarrow V_\lambda ^H(L)$ satisfying

2.8 $$\begin{aligned} f(\overline{n}hg) = \langle h \rangle _\lambda \cdot f(g) \qquad \text {for all } \overline{n} \in \overline{N}_Q(\textbf{Q}_p), h\in H(\textbf{Q}_p), g\in G(\textbf{Q}_p). \end{aligned}$$

#### Parahoric analytic coefficients

Recall $J_p \subset G(\textbf{Q}_p)$ is the *Q*-parahoric subgroup. The theory of distributions on $J_p$ was developed in [10], and—in our setting—described in detail in [12, §3]. In (2.7), we replace the second algebraic induction with locally analytic induction, i.e. we let

$$\begin{aligned} \mathcal {A}_\lambda (L) = \textrm{LAInd}_{\overline{Q}(\textbf{Z}_p)\cap J_p}^{J_p} V_\lambda ^H(L), \end{aligned}$$

whence $\mathcal {A}_\lambda (L)$ is the space of *locally analytic* $f : J_p \rightarrow V_\lambda ^H(L)$ such that

2.9 $$\begin{aligned} f(\overline{n}hg) = \langle h \rangle _\lambda \cdot f(g) \qquad \text {for all } \overline{n} \in \overline{N}_Q(\textbf{Q}_p), h\in H(\textbf{Q}_p), g\in J_p. \end{aligned}$$

Let $\mathcal {D}_\lambda (L) = {{\,\textrm{Hom}\,}}_{\textrm{cts}}(\mathcal {A}_\lambda (L),L)$ be the continuous dual.

Dualising the natural inclusion $V_\lambda (L) \subset \mathcal {A}_\lambda (L)$ yields a specialisation map

2.10 $$\begin{aligned} r_\lambda : \mathcal {D}_\lambda (L) \twoheadrightarrow V_{\lambda }^\vee (L). \end{aligned}$$

#### The $*$-action on distributions

The space $\mathcal {A}_\lambda (L)$ carries a left $J_p$-action by

$$\begin{aligned} (g * f)(h) = f(hg), \qquad h,g \in J_p. \end{aligned}$$

Recall $\varpi _{\mathfrak {p}}$ is a uniformiser for $F_{\mathfrak {p}}$, and let

2.11 $$\begin{aligned} t = t_{\mathfrak {p}} :=\iota (\varpi _{\mathfrak {p}} I_n, I_n) = \left( {\begin{smallmatrix}\varpi _{\mathfrak {p}} I_n &  \\  &  I_n\end{smallmatrix}}\right) \in {{\,\textrm{GL}\,}}_{2n}(F_{\mathfrak {p}}). \end{aligned}$$

If $f \in \mathcal {A}_\lambda$, then the function $(t_{\mathfrak {p}}^{-1} * f) : N_Q(\textbf{Z}_p) \rightarrow V_\lambda ^H(L)$ defined by $(t_{\mathfrak {p}}^{-1} * f)(n) :=f(t_{\mathfrak {p}}nt_{\mathfrak {p}}^{-1})$ extends uniquely to an element of $\mathcal {A}_\lambda$ (recalling $N_Q(\textbf{Z}_p) \subset Q(\textbf{Z}_p)$ is the unipotent radical). We get an induced dual action on $\mathcal {D}_\lambda$ given by $(t_{\mathfrak {p}} * \mu )(f) = \mu (t_{\mathfrak {p}}^{-1} * f)$, as in [12, §3.4].

Let $\Delta _p \subset G(\textbf{Q}_p)$ (resp. $\Delta _p^{-1}$) be the semigroup generated by $J_p$ and $t_{\mathfrak {p}}$ (resp. $t_{\mathfrak {p}}^{-1}$) for $\mathfrak {p}|p$. The actions above extend to actions of $\Delta _p^{-1}$ on $\mathcal {A}_\lambda$ and $\Delta _p$ on $\mathcal {D}_\lambda$.

#### Intertwining of actions

The $*$-action of $\Delta _p^{-1}$ on $\mathcal {A}_\lambda (L)$ preserves the subspace $V_\lambda (L)$. In particular, the $\Delta _p$-action on $\mathcal {D}_\lambda (L)$ descends to the quotient $V_\lambda ^\vee (L)$. Moreover, this $*$-action preserves natural integral subspaces in all of these spaces (see [12, Rem. 3.13]).

As $\Delta _p \subset G(\textbf{Q}_p)$, (2.6) induces a $\cdot$-action of $\Delta _p$ on $V_\lambda ^\vee (L)$. The $\cdot$- and $*$-actions of $J_p$ visibly agree; but the actions of $t_{\mathfrak {p}}^{-1}$ differ. From the definitions, if $\mu \in V_\lambda ^\vee (L)$, then

2.12 $$\begin{aligned} t_{\mathfrak {p}} * \mu = \lambda (t_{\mathfrak {p}}) \times (t_{\mathfrak {p}}\cdot \mu ). \end{aligned}$$

Since the $\cdot$- and $*$-actions of $J_p$ agree, if $K = K^pJ_p \subset G(\textbf{A}_f)$ is a parahoric-at-*p* level group, then both actions yield the same local system $\mathscr {V}_\lambda ^\vee (L)$ on $S_K$. However, since the actions of $t_{\mathfrak {p}}$ are different, we get two different Hecke operators on the cohomology $\textrm{H}^{\bullet }_{\textrm{c}}(S_K,\mathscr {V}_\lambda ^\vee (L))$: the ‘automorphic’ operator $U_{\mathfrak {p}}^\cdot$, which is canonical but may have non-integral eigenvalues, and the ‘*p*-adic’ operator $U_{\mathfrak {p}}^*$, which is integrally normalised but depends on the choice of $\varpi _{\mathfrak {p}}$.

### Automorphic cohomology classes

By [18, p.120], if $\pi$ has weight $\lambda$ then the $(\mathfrak {g}_\infty ,K_\infty ^\circ )$-cohomology $\textrm{H}^i(\mathfrak {g}_\infty ,K_\infty ^\circ ; \pi _\infty \otimes V_\lambda ^\vee (\textbf{C}))$ is nonzero exactly when

2.13 $$\begin{aligned} rn^2 + s(2n^2-n)\leqslant i \leqslant r(n^2+n-1) + s(2n^2+n-1). \end{aligned}$$

Via cuspidal cohomology (see [18]), for any open compact $K \subset G(\textbf{A}_f)$ there is an injective and Hecke-equivariant mapComposing with , and the isomorphism from §2.6, we get an injective map2.14

## Local test data

To get useful cohomology classes from the map (2.14), we must make good choices of local input data, that is, elements $\varphi _v \in \pi _v$ for all finite *v*, and a class $[\omega ]$ in the $(\mathfrak {g}_\infty ,K_\infty ^\circ )$-cohomology. We do so here; in §3.1 for $v\not \mid p\infty$, in §3.2 for *v*|*p*, and §3.3 at infinity.

Since our motivation is global, let $\pi$ be a RASCAR with an $(\eta ,\psi )$-Shalika model. Let $\lambda$ be the weight of $\pi$, with purity weight $\underline{\textsf{w}}\in \textbf{Z}^\Sigma$. Let $\chi \in \textrm{Crit}_p(\pi )$ be a Hecke character of infinity type $\textbf{j}$ (and *p*-power conductor). Let $V_{\chi ,s}^H$ be the character $\chi |\cdot |^s \otimes (\chi \eta |\cdot |^s)^{-1}$ of $H(\textbf{A})$. We write $V_{\chi _v,s}^H$, $V_{\chi _f,s}^H$ and $V_{\chi _\infty ,s}^H$ for the restrictions to $H(F_v)$, $H(\textbf{A}_{f})$ and $H(\textbf{R})$.

For each place *v* of *F*, fix a local intertwining $\mathcal {S}_{\psi _v}^{\eta _v}$ of $\pi _v$ into its Shalika model. This induces finite and infinite intertwinings $\mathcal {S}_{\psi _f}^{\eta _f}$ and $\mathcal {S}_{\psi _\infty }^{\eta _\infty }$.

### Friedberg–Jacquet integrals at finite places

First we recap [24, §3] (see also [31, Prop. 2.10]). Let *v* be a finite place, and fix a Haar measure $dg_v$ on ${{\,\textrm{GL}\,}}_n(F_v)$ such that ${{\,\textrm{GL}\,}}_n(\mathcal {O}_v)$ has volume 1. The *local Friedberg–Jacquet integral* is

$$\begin{aligned} Z_v(\varphi _v,\chi _v,s) :=\int _{{{\,\textrm{GL}\,}}_n(F_v)}\mathcal {S}_{\psi _v}^{\eta _v}(\varphi _v)\left[ \begin{pmatrix}g &  \\  &  1\end{pmatrix}\right] \chi _v|\cdot |^{s-\tfrac{1}{2}}\Big (\det (g)\Big ) \textrm{d}g. \end{aligned}$$

This converges absolutely for $\textrm{Re}(s) \gg 0$. Its normalisation

$$\begin{aligned} Z_v^\circ (\varphi _v,\chi _v,s) :=\frac{1}{L(\pi _v\times \chi _v,s)}Z(\varphi _v,\chi _v,s) \in \textrm{Hom}_{H(F_v)} \Big (\pi _v \otimes V_{\chi _v,s-1/2}^H, \ \textbf{C}\Big ) \end{aligned}$$

admits analytic continuation to $\textbf{C}$.

The following are proved in Propositions 3.1 and 3.2 of [24], respectively (cf. [23, Prop. 3.3]). Let $\delta _v$ be the valuation of the different of $F_v/\textbf{Q}_\ell$, where $v|\ell$, and let $q_v$ be the size of $\textbf{F}_v$.

#### Proposition 3.1

(Friedberg–Jacquet)

(i) There exists a test vector $\varphi _v^{\textrm{FJ}} \in \pi _v$ such that
  3.1 $$\begin{aligned} Z_v^\circ (\varphi _v^{\textrm{FJ}},\chi _v,s) = (q_v^{s-1/2}\chi _v(\varpi _v)^{-1})^{n\delta _v} \end{aligned}$$
  for all *s* and all unramified $\chi _v$.
(ii) If $\pi _v$ is spherical, the spherical vector is such a test vector.

Note we do *not* get 1 on the right-hand side of (3.1), as our $\psi _v$ need not have conductor $\mathcal {O}_v$.

If $\varphi _f \in \pi _f$ can be written as $\varphi _f = \otimes _v \varphi _v$, then define $Z_f^\circ (\varphi _f,\chi _f,s) :=\prod _{v\not \mid \infty } Z_v^\circ (\varphi _v,\chi _v,s)$ (and similarly $Z_f = \prod Z_v$). Note that

$$\begin{aligned} Z_f^\circ (-,\chi _f,s) \in \textrm{Hom}_{H(\textbf{A}_{F,f})}(\pi _f\otimes V_{\chi _f,s-1/2}^H, \textbf{C}). \end{aligned}$$

Let $\varphi _{f}^{\textrm{FJ},(p)} = \otimes _{v\not \mid p\infty }\varphi _v^{\textrm{FJ}} \in \pi _f^{(p)}$. If $\chi \in \textrm{Crit}_p(\pi )$ and $v \not \mid p\infty$, then $\chi _v$ is unramified, hence:

#### Proposition 3.2

For $\mathfrak {p}|p$, let $\varphi _{\mathfrak {p}} \in \pi _{\mathfrak {p}}$ be arbitrary, and let $\varphi _f = \varphi _{f}^{\textrm{FJ},(p)} \otimes (\otimes _{\mathfrak {p}|p}\varphi _{\mathfrak {p}}) \in \pi _f$. Then for any $\chi \in \textrm{Crit}_p(\pi )$, and for $\textrm{Re}(s) \gg 0$, we have

3.2 $$\begin{aligned} \textstyle Z_f(\varphi _f, \chi _f,\tfrac{1}{2}) = \left( \prod _{v\not \mid p\infty }\chi (\varpi _v^{\delta _v})^{-n}\right) \cdot \left( \prod _{\mathfrak {p}|p}Z_{\mathfrak {p}}\big (\varphi _{\mathfrak {p}},\chi _{\mathfrak {p}},\tfrac{1}{2}\big )\right) \cdot L^{(p)}\big (\pi _f\otimes \chi _f,\tfrac{1}{2}\big ), \end{aligned}$$

where $L^{(p)}$ is the *L*-function without the Euler factors at $\mathfrak {p}|p$.

### *Q*-refinements and local choices at $\mathfrak {p}|p$

For *p*-adic interpolation, it is essential to treat the primes $\mathfrak {p}|p$ separately. In this case, good local vectors have been pinned down in [23, §3], [12, §2.7], [13, §9], which we summarise here. *For ease of notation, for* §3.2*only, we will largely drop subscripts* $\mathfrak {p}$, i.e. * write* $\pi = \pi _{\mathfrak {p}}$, $\varpi = \varpi _{\mathfrak {p}}$, $J = J_{\mathfrak {p}}$, *etc.).*

Assume that $\pi$ is spherical. We write $\pi = {{\,\textrm{Ind}\,}}_B^G\theta$, with $\theta = (\theta _1,...,\theta _{2n})$ as in [13, §6.1, Notation 9.1]. In particular, we take $\theta$ such that $\theta _i\theta _{n+i} = \eta$ for $i = 1,\dots ,n$ (which is possible by [4, Prop. 1.3], since $\pi$ has an $(\eta ,\psi )$-Shalika model).

Recall $t = t_{\mathfrak {p}}$ from (2.11). On $\pi ^{J}$, we have the Hecke operator $U_{\mathfrak {p}} := [J t J]$.

#### Definition 3.3

A *Q*-*refinement* $\tilde{\pi }= (\pi ,\alpha )$ of $\pi$ is a choice of $U_{\mathfrak {p}}$-eigenvalue $\alpha$ on $\pi ^J$. We say $\tilde{\pi }$ is *regular* if $\alpha$ is a simple eigenvalue.

Recalling that $\pi = \textrm{Ind}_B^G\theta$, by [17, Lem. 4.8.4] (cf. [13, Prop. 2.5]) any *Q*-refinement is of the form

3.3 $$\begin{aligned} \alpha = q^{n^2/2}\prod _{i\in I(\tilde{\pi })}\theta _i(\varpi ), \end{aligned}$$

where $q = q_{\mathfrak {p}} :=\#\textbf{F}_{\mathfrak {p}}$ and $I(\tilde{\pi }) \subset \{1,\dots ,2n\}$ is a subset of size *n*. In particular, there are at most ${2n \atopwithdelims ()n}$ possible *Q*-refinements. Only a subset of these ‘interact well with the Shalika model’, and in practice, we are interested only in this special class of refinements.

#### Definition 3.4

Let $\tilde{\pi }= (\pi ,\alpha )$ be a regular *Q*-refinement, with corresponding subset $I(\tilde{\pi }) \subset \{1,\dots ,2n\}$ as in (3.3). We say $\tilde{\pi }$ is *spin* if $I(\tilde{\pi })$ contains exactly one of 1 or $n+1$, and exactly one of 2 or $n+2$, $\dots$, and exactly one of *n* or 2*n*. (In particular, if $I(\tilde{\pi })$ contains some $i \in \{1,\dots ,n\}$, then $I(\tilde{\pi })$ does *not* contain $n+i$.)

Note that, in the case where the ${2n \atopwithdelims ()n}$ *Q*-refinements are all different, exactly $2^n$ of them are spin. We henceforth fix a regular spin *Q*-refinement $\tilde{\pi }= (\pi ,\alpha )$. For this $\tilde{\pi }$, up to reordering the $\theta _i$, we may (and henceforth do) assume $I(\tilde{\pi }) = \{n+1,n+2,\dots ,2n\}$, whence by (3.3) we have

3.4 $$\begin{aligned} \alpha = q^{n^2/2}\theta _{n+1}(\varpi )\cdots \theta _{2n}(\varpi ). \end{aligned}$$

#### Remark 3.5

The above criterion for spin refinements was first given in [23, §3.3], where a regular spin *Q*-refinement was called ‘*Q*-regular’.

A more conceptual reinterpretation of Definition 3.4 was given in [13, Part II] and [11]. We summarise briefly. Recall $\pi$ is the functorial transfer of a representation $\Pi$ of $\textrm{GSpin}_{2n+1}(F_{\mathfrak {p}})$. Via root data, there is a natural parabolic subgroup $\mathcal {Q}\subset \textrm{GSpin}_{2n+1}$ associated with $Q\subset G$ (described in [11, §2.1]). Then $\tilde{\pi }$ is spin if and only if $\alpha$ is an eigenvalue of a Hecke operator $\mathcal {U}_{\mathfrak {p}}$ on the $\mathcal {Q}$-parahoric invariant vectors in $\Pi$; see [11, §3] for more details (noting spin here is called *Q*-spin there).

We indicate how spin refinements are expected to ‘interact well with the Shalika model’. Let $\tilde{\pi }= (\pi ,\alpha )$ be any regular *Q*-refinement, and let $W \in \mathcal {S}_{\psi }^\eta (\pi ^J)[U_{\mathfrak {p}}-\alpha ]$ be a generator of the $\alpha$-eigenspace in the Shalika model. Then we expect $W(t^{-\delta }) \ne 0$ if and only if $\tilde{\pi }$ is spin. The ‘if’ direction was proved in [23, Lem. 3.6]; the converse is predicted by [11, §8] (which also gives partial results towards this).

The expression $W(t^{-\delta })$ in the above remark is related to (twisted) local Friedberg–Jacquet integrals (see e.g. [23, Prop. 3.4]). Via a separate treatment, these twisted integrals are the subject of Proposition 3.6. They are familiar in the theory of *p*-adic *L*-functions; see, for example, §5 of the present paper.

The following ‘local Birch lemma’ is [13, Prop. 9.3]. Let $$u :=\left( {\begin{smallmatrix}1 &  w_n\\ 0 &  1\end{smallmatrix}}\right) \in {{\,\textrm{GL}\,}}_{2n}(F_{\mathfrak {p}})$$, where $w_n$ is the longest Weyl element for ${{\,\textrm{GL}\,}}_n$. Recall $\delta = \delta _{\mathfrak {p}}$ is the valuation of the different of $F_{\mathfrak {p}}$. For a smooth character $\chi$ of $F_{\mathfrak {p}}^\times$ of conductor $\mathfrak {p}^{\beta _0}\subset \mathcal {O}$, let $\tau (\chi )$ be the local Gauss sum

$$\begin{aligned} \tau (\chi ) = \tau (\chi ,\psi ) = q^{\beta _0}(1-q^{-1})\int _{\mathcal {O}^{\times }} \chi (c\varpi ^{-\beta _0-\delta }) \psi (c\varpi ^{-\beta _0-\delta }) d^{\times } c, \end{aligned}$$

where $d^\times c$ is the Haar measure on $\mathcal {O}^\times$ of total measure 1.

#### Proposition 3.6

Let $\tilde{\pi }= (\pi ,\alpha )$ be a regular spin *Q*-refinement, with $\alpha$ normalised as in (3.4). There exists an eigenvector $\varphi ^{\alpha } \in \pi ^{J}[U_{\mathfrak {p}} - \alpha ]$ such that for any smooth character $\chi$ of $F_{\mathfrak {p}}^\times$ of conductor $\mathfrak {p}^{\beta _0} \subset \mathcal {O}_{\mathfrak {p}}$, we have

$$\begin{aligned} Z_{\mathfrak {p}}\Big ((u^{-1}t^{\beta }) \cdot \varphi ^{\alpha }, \chi , \tfrac{1}{2}\Big ) = \frac{q^n}{(q-1)^n} \cdot \frac{q^{\delta (n^2-n)/2}}{\alpha ^\delta } \cdot \chi (\det w_n) \cdot Q(\pi ,\chi ), \end{aligned}$$

where $$\beta = \textrm{max}(1,\beta _0)$$ and

$$\begin{aligned} Q(\pi ,\chi )=\left\{ \begin{array}{cl} q^{\beta \left( \tfrac{-n^2-n}{2}\right) } \cdot \tau (\chi )^n & : \chi \text { ramified},\\ \displaystyle { \chi (\varpi )^{-n\delta } \cdot \alpha \cdot q^{-n^2} \cdot \prod _{i=n+1}^{2n} \frac{1-\theta _{i}^{-1}\chi ^{-1}(\varpi )q^{-1/2}}{1-\theta _{i}\chi (\varpi )q^{-1/2}}} & : \chi \text { unramified}.\end{array}\right. \end{aligned}$$

#### Proof

This is slightly rearranged from [13]; the only significant difference in the ramified case is that we exploit (3.4). In the unramified case, we have pulled a factor of $\theta _i\chi (\omega )q^{1/2}$ out of the product in $Q(\pi ,\chi )$ *op. cit.*, and again used (3.4). To make our final formula more ‘symmetric’ in $\beta$ and $\delta$, our eigenvector is $q^{\delta n^2}$ times the eigenvector from [13]. $\square$

If $\tilde{\pi }= (\pi ,\alpha )$ is not spin, then by contrast we expect this zeta integral vanishes identically on the $\alpha$-eigenspace (see [11, §8]).

### Classes at infinity

We now choose a class $[\omega ]$ at infinity, via the ‘zeta integral at infinity’. The choice connects closely to the automorphic cycles in §4 (cf. [31, §4.1]).

#### The zeta integral at infinity

Recall $\iota : H \hookrightarrow G$ and $K_\infty$ from §2.1, let $L_\infty :=H(\textbf{R}) \cap K_\infty$, and let $\mathcal {X}_H :=H(\textbf{R})^\circ /L_\infty ^\circ$. Let $\mathfrak {X}_H :=\textrm{Lie}(\mathcal {X}_H)$, and let $t :=\textrm{dim}_{\textbf{R}}(\mathfrak {X}_H)$. Letting $\mathfrak {X}_H^{\textbf{C}} :=\mathfrak {X}_H \otimes _{\textbf{R}} \textbf{C}$, we have $\wedge ^t \mathfrak {X}_H^{\textbf{C}} = \textbf{C}$. Given a $\wedge ^t\mathfrak {X}_H^{\textbf{C}}$-valued Haar measure $\mu$ on ${{\,\textrm{GL}\,}}_n(F\otimes \textbf{R})$, the attached *Friedberg–Jacquet integral at infinity*

$$\begin{aligned} Z_\mu ^\circ (-,\chi _\infty ,s) \in \textrm{Hom}_{H(\textbf{R})^\circ }\Big ( (\wedge ^t\mathfrak {X}_H^{\textbf{C}})^* \otimes \pi _\infty \otimes V_{\chi _\infty ,s-1/2}^H, \ \textbf{C}\Big ) \end{aligned}$$

is defined when $\textrm{Re}(s)\gg 0$ by

$$\begin{aligned} Z_\mu ^\circ (&\omega \otimes \varphi _\infty \otimes 1,\chi _\infty ,s) :=\\&\frac{1}{L(\pi _\infty \otimes \chi _\infty ,s)} \int _{{{\,\textrm{GL}\,}}_n(F\otimes \textbf{R})} \mathcal {S}_{\psi _\infty }^{\eta _\infty }(\varphi _\infty )\left[ \begin{pmatrix}g &  \\  &  1\end{pmatrix}\right] \chi _\infty |\cdot |^{s-\tfrac{1}{2}}\Big (\det (g)\Big ) \textrm{d}\langle \mu ,\omega \rangle g. \end{aligned}$$

#### Modular symbols at infinity

The following is [31, §4.3]. As $H(\textbf{R})^\circ$-modules, we have

$$\begin{aligned} V_{\chi _\infty }^H :=V_{\chi _\infty ,0}^H = \chi _\infty \otimes \eta _\infty ^{-1}\chi _\infty ^{-1} \cong V_{\textbf{j},-\underline{\textsf{w}}-\textbf{j}}^H(\textbf{C}). \end{aligned}$$

In particular, our choice $\kappa _{\textbf{j}} : V_\lambda ^\vee \rightarrow V^H_{\textbf{j},-\underline{\textsf{w}}-\textbf{j}}$ (from Lemma 2.6) induces an $H(\textbf{R})^\circ$-module map $V_\lambda ^\vee (\textbf{C}) \rightarrow V_{\chi _\infty }^H$ and hence an $H(\textbf{R})^\circ$-module map $V_\lambda ^\vee (\textbf{C}) \otimes (V_{\chi _\infty }^H)^\vee \rightarrow \textbf{C}$. We thus obtain a map

$$\begin{aligned} Z_\mu ^\circ (-,\chi _\infty ,1/2) \otimes \kappa _{\textbf{j}} : [\pi _\infty \otimes V_{\chi _\infty }^H] \otimes [V_\lambda ^\vee (\textbf{C})\otimes (V_{\chi _\infty }^H)^\vee ] \longrightarrow \wedge ^t\mathfrak {X}_H^{\textbf{C}}. \end{aligned}$$

##### Definition 3.7

Let $\mathfrak {h}= \textrm{Lie}(H(\textbf{R}))$. The *modular symbol at infinity* is the composition

$$\begin{aligned} \mathcal {P}_{\chi _\infty } : \textrm{H}^t(\mathfrak {g},K_\infty ^\circ ; \pi _\infty \otimes V_\lambda ^\vee (\textbf{C}))&\xrightarrow { \ \iota ^* \ } \textrm{H}^t(\mathfrak {h},L_\infty ^\circ ; \pi _\infty \otimes V_\lambda ^\vee (\textbf{C}))\\&= \textrm{H}^t(\mathfrak {h},L_\infty ^\circ ; [\pi _\infty \otimes V_{\chi _\infty }^H] \otimes [V_\lambda ^\vee (\textbf{C})\otimes (V_{\chi _\infty }^H)^\vee ])\\&\xrightarrow { \ \ Z_\mu ^\circ (-, \chi _\infty ,1/2) \otimes \kappa _{\textbf{j}}\ \ } \textrm{H}^t(\mathfrak {h},L_\infty ^\circ ;\wedge ^t\mathfrak {X}_H^{\textbf{C}}) = \textbf{C}. \end{aligned}$$

##### Theorem 3.8

(Lin–Tian, Jiang–Sun–Tian) There exists a class $[\omega ] \in \textrm{H}^t(\mathfrak {g},K_\infty ^\circ ; \pi _\infty \otimes V_\lambda ^\vee (\textbf{C}))$ such that $\mathcal {P}_{\chi _\infty }([\omega ]) \ne 0$ for all $\chi \in \textrm{Crit}(\pi )$.

##### Proof

After replacing $\pi$ (globally) with $\pi \times \widetilde{\chi }$ for some (fixed) $\widetilde{\chi } \in \textrm{Crit}(\pi )$, and modifying $\lambda$ accordingly, we may assume the weight of $\pi$ is balanced in the sense of [31] (since then $j=0$ is a critical integer). Let $[\omega ]$ be the class chosen by Jiang–Sun–Tian after Proposition 4.9 of [31]. The required non-vanishing is shown *op. cit*. for $\chi$ of the form $\chi = \chi _0|\cdot |^j$, with $\chi _0$ finite-order and $j \in \textbf{Z}$; that is, for $\textbf{j}$ parallel. If *F* has a real embedding, this accounts for all possible critical $\chi$, and we are done.

If *F* has no real embedding, then $\chi$ can have non-parallel infinity type. To see the more general non-vanishing we state here, we look at the proof in [31]. The choice of $[\omega ]$ stems from the choice of map $\phi _0$ in Lemma 5.12 *op. cit*. Once this choice is fixed, non-vanishing follows from non-vanishing of the composition of three maps in Lemma 5.13 *op. cit*. The first two maps are independent of $\chi _\infty$, so are non-vanishing as *op. cit*. Non-vanishing of the third follows from non-vanishing of $Z^\circ _\mu (-,\chi _\infty ,1/2)$ on the minimal *K*-type in $\pi _\infty$. But this was shown by Lin–Tian in [34, Thm. 1.2]. We are in situation (2) of that theorem since $\chi$ is a standard critical character. $\square$

##### Definition 3.9

Define the *period at* $\chi _\infty$ (attached to the choice $[\omega ]$) to be

3.5 $$\begin{aligned} \Omega _{\pi ,\chi _{\infty }} :=w^{\textbf{j}}(\det w_n) \cdot \mathcal {P}_{\chi _\infty }([\omega ])^{-1}, \end{aligned}$$

recalling $w_n$ is the longest Weyl element in ${{\,\textrm{GL}\,}}_n$ and $w^{\textbf{j}}$ is from §2.5.

##### Remark 3.10

The simplified (purely archimedean) statements above suffice for our purposes; but in fact, [31] prove considerably more. Using global input, one may find a $\textbf{Q}(\pi ,\eta )$-rational structure on $\textrm{H}^t(\mathfrak {g},K_\infty ^\circ ; \pi _\infty \otimes V_\lambda ^\vee (\textbf{C}))$ (see Lemma 4.7 *op. cit*.), and one may take $[\omega ]$ to be $\textbf{Q}(\pi ,\eta )$-rational. Such a rational $[\omega ]$, and the resulting period $\Omega _{\pi ,\chi _\infty }$, depends globally on $\pi$ (not just $\pi _\infty$) and is well defined up to $\textbf{Q}(\pi ,\eta )^\times$-multiple.

##### Remark 3.11

The sign $w^{\textbf{j}}(\det w_n)$ looks a little artificial and could in any case be absorbed into the choice of $\kappa _{\textbf{j}}$. It is an artefact of our later normalisation of branching laws, and we include it to allow for cleaner interpolation statements in later sections. More precisely, in Theorem 5.16 it will combine with the local terms $\chi _{\mathfrak {p}}(\det w_n)$ at $\mathfrak {p}|p$ (from Proposition 3.6), ultimately yielding a factor of $\chi _{[p]}(\det w_n)$ in the final interpolation formula. This factor can then be normalised away with a Dirac distribution; see §7.3.

Once $[\omega ]$ is fixed, $\Omega _{\pi ,\chi _{\infty }}$ depends only on the sign $\epsilon _{\chi } \in \{\pm 1\}^{\Sigma (\textbf{R})}$ (notation as in [7, §2.2.1]), and $\textbf{j}$, through the choice of branching law $\kappa _{\textbf{j}}$. We further expect that for certain normalised choices of $\kappa _{\textbf{j}}$, that $\Omega _{\pi ,\chi _{\infty }}$ should have very light, explicit dependence on $\textbf{j}$.

To be more precise: fix $\chi _0$ finite order, and let $\chi _j :=\chi _0|\cdot |^j$. In [31], the $\kappa _{\textbf{j}}$ are normalised (in Lemma 4.8) to send $$\left( {\begin{smallmatrix}1 &  -w_n\\ w_n &  1\end{smallmatrix}}\right) \cdot v_0^\vee $$ to 1, where $v_0^\vee$ is a highest weight vector in $V_\lambda ^\vee$. One of their main technical results is then that there exists $\epsilon \in \{\pm 1\}$ such that $\epsilon ^j \cdot i^{-jn[F:\textbf{Q}]} \cdot \mathcal {P}_{\chi _{j,\infty }}$ is independent of *j*. This leads to their proof of the period relations for $L(\pi \times \chi _0,s)$ as *s* ranges over critical integers *j*.

We will later, via a different method, specify choices of $\kappa _{\textbf{j}}$ via a different method that allows *p*-adic interpolation. Naturally one should expect that our choices are related to those of [31] and that such a relation should imply the value of our *p*-adic *L*-function at any special value *on the cyclotomic line* would satisfy exactly the interpolation predicted by Panchishkin (including the factor at infinity). If *F* has a real embedding, this accounts for all special values. When *F* has no real embedding, it is natural to hope that an adaptation of the methods in [31] would provide an analogous result for the more general $\mathcal {P}_{\chi _\infty }$, which would mean our *p*-adic *L*-functions have the correct interpolation factor at $\infty$ at *all* special values.

### Summary

We collect together our choices.

#### Test Data 3.12

Let $\pi$ be a RASCAR of weight $\lambda$ spherical at every $\mathfrak {p}|p$. Then:

- For each $\mathfrak {p}|p$, fix a regular spin *Q*-refinement $\tilde{\pi }_{\mathfrak {p}} = (\pi _{\mathfrak {p}},\alpha _{\mathfrak {p}})$ of $\pi _{\mathfrak {p}}$. Write $\tilde{\pi }= (\pi , \alpha )$ for the collection of these choices, for $\alpha = (\alpha _{\mathfrak {p}})_{\mathfrak {p}|p}$. Let $\varphi _{\mathfrak {p}}^{\alpha _{\mathfrak {p}}} \in \pi _{\mathfrak {p}}$ be as in Proposition 3.6.
- Away from *p*, let $\varphi _{f}^{\textrm{FJ},(p)} = \otimes _{v\not \mid p\infty }\varphi _v^{\textrm{FJ}} \in \pi _f^{(p)}$, for $\varphi _v^{\textrm{FJ}}$ as in Proposition 3.1.
- At $\infty$, let $[\omega ] \in \textrm{H}^t(\mathfrak {g},K_\infty ^\circ ; \pi _\infty \otimes V_\lambda ^\vee (\textbf{C}))$ be the class from Theorem 3.8.

Write $$\varphi _f^{\textrm{FJ},\alpha } = \varphi _f^{\textrm{FJ},(p)} \otimes (\otimes _{\mathfrak {p}|p}\varphi _{\mathfrak {p}}^{\alpha _{\mathfrak {p}}})$$.

Let $u^{-1}t_p^{\beta } = \prod _{\mathfrak {p}|p}u^{-1}t_{\mathfrak {p}}^{\beta _{\mathfrak {p}}} \in G(\textbf{Q}_p)$. Then for any $\chi \in \textrm{Crit}_p(\pi )$, we have computed $\mathcal {P}_{\chi _\infty }([\omega ]) \cdot Z_f^\circ (u^{-1}t_p^\beta \cdot \varphi _f^{\textrm{FJ},\alpha }, \chi , 1/2)$ in terms of $L(\pi \times \chi ,1/2)$, explicit terms at *p*, and a *nonzero* period.

## Critical *L*-values via cohomology

We now describe a cohomological interpretation of the Friedberg–Jacquet integrals, and hence the standard critical *L*-values, following [27] and [31]. We begin to tailor our approach towards *p*-adic interpolation, however, with features from [12, 13, 23].

Throughout, let $\pi$ be a RASCAR with an $(\eta ,\psi )$-Shalika model. Let $\lambda$ be the weight of $\pi$, with purity weight $\underline{\textsf{w}}$. Let $\chi \in \textrm{Crit}_p(\pi )$ be an algebraic Hecke character of infinity type $\textbf{j}$ and conductor $p^{\beta _0}= \prod _{\mathfrak {p}|p}\mathfrak {p}^{\beta _{0,\mathfrak {p}}}$, where $\beta _0 = (\beta _{0,\mathfrak {p}}) \in \textbf{Z}_{\geqslant 0}^{\mathfrak {p}|p}$ is a multiexponent. For technical reasons (cf. Proposition 3.6), we let $\beta _{\mathfrak {p}} = \max (1,\beta _{0,\mathfrak {p}})$ and let $\beta = (\beta _{\mathfrak {p}}) \in \textbf{Z}_{\geqslant 1}^{\mathfrak {p}|p}$.

### Automorphic cycles

One of the main results of [24, §2], reinterpreted in [31, Prop. 4.1], is that the product

$$\begin{aligned} Z_\mu ^\circ \cdot Z_f^\circ (-,\chi ,s) : (\wedge ^t \mathfrak {X}_H^{\textbf{C}})^* \otimes \pi \otimes V_{\chi ,s-1/2}^H \longrightarrow \textbf{C}, \end{aligned}$$

introduced here in §3, can be computed via period integrals for $H \subset G$. In particular, they showed that there exists a Haar measure $\mu$ such that

$$\begin{aligned} [Z_\mu ^\circ (-,\chi _\infty ,s) \cdot Z_f^\circ (-,\chi _f,s)\big ](\omega \otimes \varphi \otimes 1) \cdot L(\pi \times \chi ) = \int _{X_\beta } \varphi \cdot \omega , \end{aligned}$$

where $X_\beta$ is an *automorphic cycle* for *H* that we define below. We will explain this and interpret the right-hand side via cohomology.

For the rest of §4, let $K \subset G(\textbf{A}_f)$ be an open compact subgroup fixing $\varphi _f^{\textrm{FJ},\alpha }$ from Test Data 3.12. Recall $L_\infty = H(\textbf{R}) \cap K_\infty$. (Here we take intersections with *H* with respect to $\iota$.)

#### Definition 4.1

Let $L \subset H(\textbf{A}_f) \cap K$ be open compact. The *automorphic cycle of level* *L* is

$$\begin{aligned} X_L :=H(\textbf{Q})\backslash H(\textbf{A}) /L L_\infty ^\circ . \end{aligned}$$

By [3, Lemma 2.7], $\iota : H \hookrightarrow G$ induces a proper map $X_L \hookrightarrow S_K$, which we also denote $\iota$.

In §5, we will choose $L_\beta \subset H(\textbf{A}_f)$ at $\beta$, which (as in [12, §4.1]) is sufficiently small that:

- $\chi$ and $\eta$ are trivial on $L_\beta$,
- $X_\beta :=X_{L_\beta }$ is a real manifold,
- and $H(\textbf{Q}) \cap hL_\beta L^\circ _\infty h^{-1} = Z_G(\textbf{Q}) \cap L_\beta L_\infty ^\circ$ for all $h \in H(\textbf{A})$.

Recall $t = \textrm{dim}_{\textbf{R}}(\mathcal {X}_H) = \textrm{dim}_{\textbf{R}}(X_\beta )$, and *F* has *r* (resp. *s*) real (resp. complex) places.

#### Lemma 4.2

We have $$t = \textrm{dim}_{\textbf{R}}(X_\beta ) = r(n^2+n-1) + s(2n^2-1).$$

#### Proof

At each real place, we get a contribution of $[{{\,\textrm{GL}\,}}_n(\textbf{R})\times {{\,\textrm{GL}\,}}_n(\textbf{R})]/[\textrm{O}_n(\textbf{R})\times \textrm{O}_n(\textbf{R})]\textbf{R}^\times$, which has dimension $2(n^2) - 2(n^2-n)/2 - 1 = n^2 + n - 1$.

At each complex place, we get $[{{\,\textrm{GL}\,}}_n(\textbf{C})\times {{\,\textrm{GL}\,}}_n(\textbf{C})]/[\textrm{U}_n(\textbf{C}) \times \textrm{U}_n(\textbf{C})]\textbf{C}^\times$, which has dimension $2(2n^2) - [2(n^2) + 2 - 1] = 2n^2-1$, noting $\textrm{dim}_{\textbf{R}}(\textbf{C}^\times \cap [\textrm{U}_n(\textbf{C})\times \textrm{U}_n(\textbf{C})]) = 1$. $\square$

#### Remark 4.3

Recall the supported range of cuspidal cohomology degrees

$$\begin{aligned}rn^2 + s(2n^2-n) \leqslant i \leqslant r(n^2+n-1) + s(2n^2+n-1)\end{aligned}$$

from (2.13). We see that at each real place, there is a contribution of $n^2+n-1$ to the dimension of automorphic cycles, exactly at the top of the range $[n^2, n^2+n-1]$. For complex places, the contribution of $2n^2-1$ is just below the middle of the range $[2n^2-n, 2n^2+n-1]$.

### Evaluation maps, I

Still summarising [31], we now give a global version. Recall $\kappa _{\textbf{j}} : V_\lambda ^\vee \rightarrow V_{\textbf{j},-\underline{\textsf{w}}-\textbf{j}}^H$ from Lemma 2.6. Let $\textrm{Ev}_{\textbf{j}}^{\textrm{JST}}$ be the composition4.1The final arrow is an integration map $\int _{X_\beta }$ (that we will make precise in (5.4)).

As in [31, (4.12)], the Lie algebra cohomology yields a natural injective map (and class)The choice of $[\omega ]$ (from Theorem 3.8) yields, via (2.14), an injective map4.2The following is the commutative diagram after [31, Lem. 4.11]. The term $\textrm{vol}(L_\beta )$ arises because of our normalisation of $\int _{X_\beta }$, compared to their period integral in (4.7) *op. cit*.

#### Proposition 4.4

We have a commutative diagram

Finally, we rephrase in language we will consider in the next sections, and summarise all the above results. Let

4.3 $$\begin{aligned} \textrm{Ev}_{\chi }^{\textrm{JST}} : \textrm{H}^{t}_{\textrm{c}}(S_K,\mathcal {V}_\lambda ^\vee (\textbf{C})) \longrightarrow \textbf{C}, \qquad \phi \longmapsto \textrm{Ev}_{\textbf{j}}^{\textrm{JST}}\big (\phi \otimes [V_{\chi _f}^H]\big ). \end{aligned}$$

From Proposition 3.2, we see immediately that:

#### Corollary 4.5

If $\varphi _f = \varphi _f^{\textrm{FJ},(p)} \otimes (\otimes _{\mathfrak {p}|p}\varphi _{\mathfrak {p}}) \in \pi _f^K$, then for all $\chi \in \textrm{Crit}(\pi )$ with infinity type $\textbf{j}$, we have

4.4 $$\begin{aligned} \textrm{Ev}_{\chi }^{\textrm{JST}}\circ \Theta _{[\omega ]}^K(\varphi _f) = \frac{w^{\textbf{j}}(\det w_n) }{\textrm{vol}(L_\beta )} \cdot \prod _{v\not \mid p\infty }\chi (\varpi _v^{\delta _v})^{-n}\cdot \prod _{\mathfrak {p}|p}Z_{\mathfrak {p}}\big (\varphi _{\mathfrak {p}},\chi _{\mathfrak {p}},\tfrac{1}{2}\big ) \cdot \frac{L^{(p)}\big (\pi \times \chi ,\tfrac{1}{2}\big )}{\Omega _{\pi ,\chi _{\infty }}}. \end{aligned}$$

Here the term $w^{\textbf{j}}(\det w_n)$ is coming from (3.5).

## Evaluation maps

We now give abstract generalisations of $\textrm{Ev}_{\chi }^{\textrm{JST}}$. To connect the above to the study in [12], we first give a more pedestrian reinterpretation of $\textrm{Ev}_{\chi }^{\textrm{JST}}$, before describing analogues with *p*-*adic* local systems. As in the last section, $\pi$ will be a RASCAR with an $(\eta ,\psi )$-Shalika model, of weight $\lambda$, with purity weight $\underline{\textsf{w}}$. Let $\chi$ be an algebraic Hecke character of conductor $p^{\beta _0}$, with $\beta _0 = (\beta _{0,\mathfrak {p}})_{\mathfrak {p}|p} \in \textbf{Z}^{\mathfrak {p}|p}_{\geqslant 0}$, and infinity type $\textbf{j}$ critical for $\pi$. Again set $\beta _{\mathfrak {p}} = \max (1,\beta _{0,\mathfrak {p}})$ and let $\beta = (\beta _{\mathfrak {p}}) \in \textbf{Z}_{\geqslant 1}^{\mathfrak {p}|p}$.

We want to work with *p*-adic, rather than complex, coefficients; so recall we fixed an isomorphism $i_p : \textbf{C}\rightarrow \overline{\textbf{Q}}_p$. Via this isomorphism, we identify $V_\lambda ^\vee (\textbf{C})$ and $V_\lambda ^\vee (\overline{\textbf{Q}}_p)$, and let5.1for $\Theta _{[\omega ]}^K$ the map from (4.2).

### Automorphic cycles revisited

#### Level structures

We now specify the levels $L_\beta$. Let $K = \prod _v K_v \subset G(\textbf{A}_f)$, such that:

(i) For $v \not \mid p\infty$, we take $K_v$ fixing $\varphi _v^{\textrm{FJ}}$ (from §3.1);
(ii) For $\mathfrak {p}|p$, we take $K_{\mathfrak {p}} = J_{\mathfrak {p}}$ parahoric (as in §2.1).

Let $u \in G(\textbf{A}_f)$ be the matrix with $u_{v} = 1$ if $v\not \mid p$, and $$u_{\mathfrak {p}} = \left( {\begin{smallmatrix}1 &  w_n\\ 0 &  1\end{smallmatrix}}\right) $$ for all $\mathfrak {p}|p$. For a multiexponent $\beta = (\beta _{\mathfrak {p}})_{\mathfrak {p}|p}$, let $L_\beta = L^{(p)} \prod _{\mathfrak {p}|p} L_{\mathfrak {p}}^{\beta _{\mathfrak {p}}} \subset H(\textbf{A}_f)$, where (taking all intersections with *H* with respect to $\iota$):

(i) away from *p*, $L^{(p)} \subset G(\textbf{A}_f^{(p)})$ is the principal congruence subgroup of some (fixed, suppressed) prime-to-*p* ideal $\mathfrak {m}\subset \mathcal {O}_F$, chosen so that $L^{(p)} \subset H(\textbf{A}_f^{(p)}) \cap K^{(p)}$;
(ii) and $$L_{\mathfrak {p}}^{\beta _{\mathfrak {p}}} = H(\textbf{Z}_p) \cap K_{\mathfrak {p}} \cap \Big (u_{\mathfrak {p}}^{-1}t_{\mathfrak {p}}^{\beta _{\mathfrak {p}}} \cdot K_{\mathfrak {p}} \cdot t_{\mathfrak {p}}^{-\beta _{\mathfrak {p}}}u_{\mathfrak {p}} \Big ).$$

We take $\mathfrak {m}$ large enough that the three conditions after Definition 4.1 are satisfied, and as *op. cit.*, we let $X_\beta :=X_{L_\beta }$, the *automorphic cycle of level* $p^\beta$.

##### Remark 5.1

The matrix $u_{\mathfrak {p}}$ is an open-orbit representative of the spherical variety *G*/*H* (that is, $B(F_{\mathfrak {p}}) \cdot u_{\mathfrak {p}} \cdot H(F_{\mathfrak {p}})$ is open in $G(F_{\mathfrak {p}})$). The matrix $t_{\mathfrak {p}}$ induces the action of the $U_{\mathfrak {p}}$ Hecke operator.

#### Connected components and fundamental classes

##### Lemma 5.2

The map $(\det _1/\det _2, 1/\det _2)$ descends to a surjective map

##### Proof

Identical to [23, Lem. 2.1] and [13, Lem. 4.5]. $\square$

By strong approximation for *H*, the connected components of $X_\beta$ are indexed by

5.2 $$\begin{aligned} \pi _0(X_\beta ) :=\textrm{Cl}_F^+(p^\beta \mathfrak {m}) \times \textrm{Cl}_F^+( \mathfrak {m}), \end{aligned}$$

where $\textrm{Cl}_F^+(I)$ is the narrow ray class group of conductor *I*. For $\delta \in H(\textbf{A}_f)$, we write $[\delta ]$ for its associated class in $\pi _0(X_\beta )$ and denote the corresponding connected component

$$\begin{aligned} X_\beta [\delta ] :=H(\textbf{Q})\backslash H(\textbf{Q})\delta L_\beta H(\textbf{R})^\circ /L_\beta L_\infty ^\circ . \end{aligned}$$

We can describe these components as arithmetic quotients of the symmetric space $\mathcal {X}_H = H(\textbf{R})^\circ /L_\infty ^\circ$ from §3.3. For $\beta \in \textbf{Z}_{\geqslant 1}$ and $\delta \in H(\textbf{A}_f)$, define a congruence subgroup

5.3 $$\begin{aligned} \Gamma _{\beta ,\delta } :=H(\textbf{Q}) \cap \delta L_\beta H(\textbf{R})^\circ \delta ^{-1}. \end{aligned}$$

The group $\Gamma _{\beta ,\delta }$ acts on $\mathcal {X}_H$ by left translation via $\Gamma _{\beta ,\delta } \hookrightarrow H(\textbf{R})^\circ$, and there is an isomorphismwhere if $[h_\infty ] \in \mathcal {X}_H$, we write $[h_\infty ]_\delta$ for its image in $\Gamma _{\beta ,\delta }\backslash \mathcal {X}_H$.

Since $\Gamma _{\beta ,\delta }\backslash \mathcal {X}_H$ is a connected orientable manifold of dimension *t*, there is an isomorphism

$$\begin{aligned} \textrm{H}_t^{\textrm{BM}}(\Gamma _{\beta ,\delta }\backslash \mathcal {X}_H,\textbf{Z}) \cong \textbf{Z}. \end{aligned}$$

A *fundamental class* $\theta _\delta \in \textrm{H}_t^{\textrm{BM}}(\Gamma _{\beta ,\delta }\backslash \mathcal {X}_H,\textbf{Z})$ is a generator of this group; choosing such a class is equivalent to choosing an orientation on $\Gamma _{\beta ,\delta }\backslash \mathcal {X}_H$. Following the process described in [23, §2.2.5] and [12, §4.2.3], we choose a family $(\theta _\delta )_\delta$ of such classes compatibly as $\delta$ (and $\beta$) varies. Choose, once and for all, an ordered basis of the tangent space of the symmetric space $H_\infty ^\circ /L_\infty ^\circ$, which induces—for any $\beta$—a fundamental class $\theta [\beta ]$ for $X_\beta [1] \subset X_\beta$, the connected component of the identity. For any $\delta$, let $\theta _{[\delta ]} = \delta _*\theta [\beta ]$, which is a fundamental class for $X_\beta [\delta ]$. Then we finally define $\theta _\delta = c_\delta ^*\theta _{[\delta ]}$.

For each $\delta$, the choice of $\theta _\delta$ yields an isomorphismOur choices, and the maps $c_\delta$, induce an integration map promised in (4.1):

5.4 $$\begin{aligned} \int _{X_\beta } :=\sum _\delta (-\cap \theta _\delta ) \circ c_\delta ^* : \textrm{H}^{t}_{\textrm{c}}(X_\beta ,\textbf{Z}) \longmapsto \textbf{Z}\end{aligned}$$

### The map $\textrm{Ev}_{\chi }^{\textrm{JST}}$ revisited

Now let $\textbf{Q}_p\subset L \subset \overline{\textbf{Q}}_p$ containing $\textbf{Q}(\pi ,\eta )$. For *p*-adic interpolation, we must twist the map $\iota : X_\beta \rightarrow S_K$. Define

$$\begin{aligned} \iota _\beta : X_\beta \longrightarrow S_K, \qquad [h] \mapsto [hu^{-1}t_p^{\beta }], \qquad h \in H(\textbf{A}), \ \ t_p = \prod _{\mathfrak {p}|p}t_{\mathfrak {p}}^{\beta _{\mathfrak {p}}}. \end{aligned}$$

One may check $\iota _\beta ^*\mathcal {V}_\lambda ^\vee = \iota ^*\mathcal {V}_\lambda ^\vee$, so for $\delta \in H(\textbf{A})$, we have maps

$$\begin{aligned} \textrm{H}^{t}_{\textrm{c}}(S_K,\mathcal {V}_\lambda ^\vee (L)) \xrightarrow { \ \iota _\beta ^* \ } \textrm{H}^{t}_{\textrm{c}}(X_\beta ,\iota ^*\mathcal {V}_\lambda ^\vee (L)) \xrightarrow { \ c_\delta ^* \ } \textrm{H}^{t}_{\textrm{c}}(\Gamma _{\beta ,\delta }\backslash \mathcal {X}_H, c_\delta ^*\iota ^*\mathcal {V}_{\lambda }^\vee (L)), \end{aligned}$$

where $c_\delta ^*\iota ^*\mathcal {V}_\lambda ^\vee$ can be checked to be the local system given by locally constant sections of $\Gamma _{\beta ,\delta }\backslash [\mathcal {X}_H \times V_\lambda ^\vee (L)] \rightarrow \Gamma _{\beta ,\delta }\backslash \mathcal {X}_H$ with action $\gamma ([h_\infty ],v) = ([\gamma h_\infty ], \gamma \cdot v)$ (cf. §2.6).

If *M* is a left $\Gamma _{\beta ,\delta }$-module, let $M_{\Gamma _{\beta ,\delta }} :=M/\{m - \gamma \cdot m : m \in M, \gamma \in \Gamma _{\beta ,\delta }\}$ be the coinvariants. The quotient map $V_\lambda ^\vee (L) \rightarrow (V_{\lambda }^\vee (L))_{\Gamma _{\beta ,\delta }}$ trivialises the local system, inducing a map

$$\begin{aligned} \textrm{coinv}_{\beta ,\delta } : \textrm{H}^{t}_{\textrm{c}}(\Gamma _{\beta ,\delta }\backslash \mathcal {X}_H, c_\delta ^*\iota ^*\mathcal {V}_{\lambda }^\vee (L)) \longrightarrow \textrm{H}^{t}_{\textrm{c}}(\Gamma _{\beta ,\delta }\backslash \mathcal {X}_H, \textbf{Z}) \otimes _{\textbf{Z}} (V_{\lambda }^\vee (L))_{\Gamma _{\beta ,\delta }}. \end{aligned}$$

#### Definition 5.3

Define5.55.6

Recall $V^H_{\textbf{j},-\underline{\textsf{w}}-\textbf{j}}$ is the algebraic *H*-representation of highest weight $(\textbf{j},-\underline{\textsf{w}}-\textbf{j})$. Recalling $w_p^{\textbf{j}}$ from (2.3), $V^H_{\textbf{j},-\underline{\textsf{w}}-\textbf{j}}(L)$ is an $H(\textbf{Q}_p)$-representation, with $h =(h_1,h_2) \in H(\textbf{Q}_p)$ acting by5.7Recall we chose (via Lemma 2.6) a nonzero element $\kappa _{\textbf{j}} \in {{\,\textrm{Hom}\,}}_{H(\textbf{Q}_p)}(V_\lambda ^\vee (L),V^H_{\textbf{j},-\underline{\textsf{w}}-\textbf{j}}(L))$.

#### Lemma 5.4

The map $\kappa _{\textbf{j}}$ induces a map $\kappa _{\textbf{j}} : (V_\lambda ^\vee )_{\Gamma _{\beta ,\delta }} \rightarrow V^H_{\textbf{j},-\underline{\textsf{w}}-\textbf{j}}(L)$.

#### Proof

It suffices to prove $\Gamma _{\beta ,\delta }$ acts trivially on the target. If $\gamma = (\gamma _1,\gamma _2) \in \Gamma _{\beta ,\delta }$, then

$$\begin{aligned} \det (\gamma _1),\det (\gamma _2) \in \mathcal {O}_{F,+}^\times . \end{aligned}$$

Moreover, $\det (\gamma _1/\gamma _2) \equiv 1 \hspace{2pt}(\textrm{mod}\hspace{2pt}p^\beta )$ and $\det (\gamma _2) \equiv 1 \hspace{2pt}(\textrm{mod}\hspace{2pt}\mathfrak {m})$ (by Lemma 5.2).

By assumption, there exists an algebraic Hecke character $\chi$ of infinity type $\textbf{j}$ and conductor dividing $p^\beta$; this forces $w_p^{\textbf{j}}(\det (\gamma _1/\gamma _2)) = w^{\textbf{j}}(\det (\gamma _1/\gamma _2)) = 1$, as in [7, §5.2.3]. Similarly, existence of $\eta$ with infinity type $\underline{\textsf{w}}$ and conductor dividing $\mathfrak {m}$ forces $w_p^{-\underline{\textsf{w}}}(\det (\gamma _2)) = 1$. Thus, $(w_p^{\textbf{j}}\otimes w_p^{-\underline{\textsf{w}}-\textbf{j}})(\gamma ) = 1$, i.e. $\gamma$ acts trivially on $V^H_{\textbf{j},-\underline{\textsf{w}}-\textbf{j}}(L)$, as required. $\square$

#### Definition 5.5

Let

$$\begin{aligned} \textrm{Ev}_{\chi } : \textrm{H}^{t}_{\textrm{c}}(S_K,\mathcal {V}_\lambda ^\vee (L))&\longrightarrow L(\chi ), \\ \phi&\longmapsto \sum _{\delta \in \pi _0(X_\beta )} (\chi \otimes \chi ^{-1}\eta ^{-1})(\delta ) \cdot \Big [\kappa _{\textbf{j}}\circ \textrm{Ev}_{\beta ,\delta }(\phi )\Big ]. \end{aligned}$$

Here we have chosen a basis $u_{\textbf{j}}$ of $V_{\textbf{j},-\underline{\textsf{w}}-\textbf{j}}^H(L)$ to identify it with $L(\chi )$. Via $i_p$, we can do this compatibly with the choices in §4.2.

By considering how all these maps behave on the local systems, we see that actually this ‘new’ construction is just a twisted version of the map $\textrm{Ev}_{\chi }^{\textrm{JST}}$ from (4.3) (hence it is independent of the choices of $\delta$). Precisely, recall

$$\begin{aligned} u^{-1}t_p^\beta = \textstyle \prod _{\mathfrak {p}|p}u^{-1}t_{\mathfrak {p}}^{\beta _{\mathfrak {p}}} \subset G(\textbf{Q}_p), \qquad \text { and let } \qquad K_\beta :=u^{-1}t_p^\beta K t_p^{-\beta } u. \end{aligned}$$

Note $L_\beta \subset K_\beta$, so we can apply $\textrm{Ev}_{\chi }^{\textrm{JST}}$ at level $K_\beta$. If $\varphi _f \in \pi ^K$, then $u^{-1}t_p^\beta \cdot \varphi _f \in \pi _f^{K_\beta }$, and:

#### Lemma 5.6

The following diagram commutes:

Note that the twisting matrix $u^{-1}t_p^\beta$ is exactly what appeared in Proposition 3.6.

### Abstract evaluation maps

So far we have only considered evaluation maps only with coefficients in $\mathcal {V}_\lambda ^\vee$, the local system attached to $V_\lambda ^\vee$ with its $G(\textbf{Q})$-action. Ultimately we will consider coefficients in *p*-adic distributions, for which only the *K*-local systems make sense. We now present a version of the above maps for *K*-local systems, with abstract coefficient modules, generalising those constructed in [12, §4] (when *F* is totally real) and [7, §10.1] (for ${{\,\textrm{GL}\,}}_2$).

The constructions/proofs of [12] all go through exactly as *op. cit*., so we omit details.

Recall $\Delta _p \subset G(\textbf{Q}_p)$ from §2.7.3. Let *M* be a left $\Delta _p$-module, with action denoted $\bullet$. (When $M = \mathcal {D}_\lambda$, this will be the $*$-action. When $M = V_\lambda ^\vee$, recall from §2.7.4 that we have two such actions, the $\cdot$- and $*$-actions, and we shall need to consider both).

The level *K* acts on *M* via its projection to $J_p = \prod _{\mathfrak {p}|p}J_{\mathfrak {p}} \subset \Delta _p$, giving a local system $\mathscr {M}$ on $S_K$ via §2.6. If $\gamma \in \Gamma _{\beta ,\delta }$ (from (5.3)), then $\delta ^{-1}\gamma \delta \in L_\beta \subset K$, so $\Gamma _{\beta ,\delta }$ acts on *M* via $\gamma \bullet _{\Gamma _{\beta ,\delta }}m :=(\delta ^{-1}\gamma \delta )_f\bullet m$. Hence, we may take coinvariants for this action.

The evaluation maps for $\mathscr {M}$ are inspired by those in Definition 5.3 for $\mathcal {V}_\lambda ^\vee$. A crucial difference is that $\iota _\beta ^*\mathscr {M}$ is *not* $\iota ^*\mathscr {M}$ any more, but there is a map

$$\begin{aligned} \tau _\beta ^\bullet : \iota _\beta ^*\mathscr {M}\rightarrow \iota ^*\mathscr {M}, \qquad (h,m) \mapsto (h,u^{-1}t_p^\beta \bullet m). \end{aligned}$$

#### Definition 5.7

The *evaluation map for* $(M,\bullet )$ *of level* $p^\beta$ at $\delta$ is the composition5.8

We track dependence on $M,\delta ,\beta$. The following are proved exactly as in [12, §4.3].

#### Lemma 5.8

(Variation in *M*; [12, Lem. 4.6]) Let $\kappa : M \rightarrow N$ be a $\Delta _p$-module map. There is a commutative diagram

#### Proposition 5.9

(Variation in $\delta$; [12, Prop. 4.9]) Let *N* be a left $H(\textbf{A})$-module, with action $\bullet$, such that $H(\textbf{Q})$ and $H(\textbf{R})^\circ$ act trivially. Let $\kappa : M\rightarrow N$ be a map of $L_\beta$-modules. Then

$$\begin{aligned} \mathscr {E}\hspace{-1pt}{\scriptstyle \mathscr {V}}_{\beta ,[\delta ]}^{M,[\bullet ],\kappa } :=\delta \bullet \left[ \kappa \circ \mathscr {E}\hspace{-1pt}{\scriptstyle \mathscr {V}}_{\beta ,\delta }^{M, [\bullet ]}\right] : \textrm{H}^{t}_{\textrm{c}}(S_K,\mathscr {M}) \longrightarrow N \end{aligned}$$

is well defined and independent of the representative $\delta$ of $[\delta ]$.

Fix $\mathfrak {p}|p$, and define $\beta ' = (\beta _\mathfrak {q}')_{\mathfrak {q}|p}$, where $\beta _{\mathfrak {p}}' = \beta _{\mathfrak {p}} + 1$ and $\beta _\mathfrak {q}' = \beta _\mathfrak {q}$ for $\mathfrak {q} \ne \mathfrak {p}$. We have natural projections

$$\begin{aligned} \textrm{pr}_{\beta ,\mathfrak {p}} : X_{\beta '} \longrightarrow X_\beta , \ \ \ \textrm{pr}_{\beta ,\mathfrak {p}} : \pi _0(X_{\beta '}) \rightarrow \pi _0(X_\beta ). \end{aligned}$$

The action of $t_{\mathfrak {p}} \in \Delta _p$ on *M* yields an action of $U_{\mathfrak {p}}^\bullet :=[Kt_{\mathfrak {p}}K]$ on $\textrm{H}^{t}_{\textrm{c}}(S_K,\mathscr {M})$, via [12, §2.3.2].

#### Proposition 5.10

(Variation in $\beta$; [12, Prop. 4.10]) Let *N* and $\kappa$ be as in Proposition 5.9. If $\beta > 0$, then as maps $\textrm{H}^{t}_{\textrm{c}}(S_K,\mathscr {M}) \rightarrow N$ we have

$$\begin{aligned} \sum _{[\eta ] \in \textrm{pr}_{\beta ,\mathfrak {p}}^{-1}([\delta ])} \mathscr {E}\hspace{-1pt}{\scriptstyle \mathscr {V}}_{\beta ',[\eta ]}^{M,[\bullet ],\kappa } = \mathscr {E}\hspace{-1pt}{\scriptstyle \mathscr {V}}_{\beta ,[\delta ]}^{M,[\bullet ],\kappa } \circ U_{\mathfrak {p}}^\bullet . \end{aligned}$$

### Classical evaluation maps

We now use the above to construct linear functionals

$$\begin{aligned} \mathscr {E}\hspace{-1pt}{\scriptstyle \mathscr {V}}_{\chi }^{[\bullet ]} : \textrm{H}^{t}_{\textrm{c}}(S_K,\mathscr {V}_\lambda ^\vee (L)) \longrightarrow L(\chi ), \end{aligned}$$

analogous to $\textrm{Ev}_\chi$ with $\mathcal {V}_\lambda ^\vee$. We have

$$\begin{aligned} \mathscr {E}_{\beta ,\delta }^{V_\lambda ^\vee ,[\bullet ]} : \textrm{H}^{t}_{\textrm{c}}(S_K,\mathscr {V}_\lambda ^\vee (L)) \longrightarrow (V_\lambda ^\vee (L))_{\Gamma _{\beta ,\delta }}. \end{aligned}$$

Lemma 5.4 says $\kappa _{\textbf{j}}$ factors through the $\Gamma _{\beta ,\delta }$-coinvariants. That lemma used the $\cdot$-action on $V_\lambda ^\vee$, but by the same proof it is also true of the $*$-action.

To apply Proposition 5.9 to $\kappa _{\textbf{j}}$, we now extend $V^H_{\textbf{j},-\underline{\textsf{w}}-\textbf{j}}(L)$ to an $H(\textbf{A})$-module in such a way that $H(\textbf{Q})$ and $H(\textbf{R})^\circ$ act trivially (as required by the statement of that proposition).

#### Definition 5.11

Let $V_{\chi ,[p]}^H(L)$ be the 1-dimensional $H(\textbf{A})$-module $\chi _{[p]}\otimes \chi _{[p]}^{-1}\eta _{[p]}^{-1}$, recalling $\chi _{[p]}$ and $\eta _{[p]}$ are the ray class characters attached to $\chi$ and $\eta$ in §2.5. Precisely, it is the space $L(\chi )$, with $h = (h_1,h_2) \in H(\textbf{A})$ acting as

$$\begin{aligned} h\cdot v = \Big [\chi _{[p]}\left( \textrm{det}\left( \tfrac{h_1}{h_2}\right) \right) \cdot \eta _{[p]}\left( \textrm{det}\left( \tfrac{1}{h_2}\right) \right) \Big ] v. \end{aligned}$$

By construction, $H(\textbf{Q})$ and $H(\textbf{R})^\circ$ act trivially on $V_{\chi ,[p]}^H$.

#### Lemma 5.12

The identity map is an isomorphism of 1-dimensional $L_\beta$-modulesHere $L_\beta$ acts on $V^H_{\textbf{j},-\underline{\textsf{w}}-\textbf{j}}$ via projection to $H(\textbf{Q}_p)$, and on $V_{\chi ,[p]}^H$ by restricting the $H(\textbf{A})$-action.

#### Proof

Via (5.7), $\ell = (\ell _1,\ell _2) \in L_\beta$ acts on $V^H_{\textbf{j},-\underline{\textsf{w}}-\textbf{j}}(L)$ as

5.9 $$\begin{aligned} \ell \cdot v = (w_p^{\textbf{j}}\otimes w_p^{-\underline{\textsf{w}}-\textbf{j}})(\ell _p) \ v. \end{aligned}$$

By definition of $\chi _{[p]}$ and $\eta _{[p]}$, the action on $V_{\chi ,[p]}^{H}$ is by

5.10 $$\begin{aligned} \ell \cdot v = \chi (\ell _1\ell _2^{-1}) \eta (\ell _2^{-1})\times (w_p^{\textbf{j}}\otimes w_p^{-\underline{\textsf{w}}-\textbf{j}})(\ell _p) \ v. \end{aligned}$$

Now $\det (\ell _1\ell _2^{-1}) \equiv 1\hspace{2pt}(\textrm{mod}\hspace{2pt}p^\beta \widehat{\mathcal {O}}_F)$ by Lemma 5.2, so as $\chi$ has conductor dividing $p^\beta$, we see $\chi (\ell _1\ell _2^{-1}) :=\chi (\det (\ell _1\ell _2^{-1})) = 1$. Similarly $\det (\ell _2) \equiv 1 \hspace{2pt}(\textrm{mod}\hspace{2pt}\mathfrak {m})$, and the conductor of $\eta$ divides $\mathfrak {m}$, we have $\eta (\ell _2^{-1}) = 1$. In particular, (5.9) and (5.10) agree, proving the lemma. $\square$

#### Corollary 5.13

For any $\delta \in H(\textbf{A}_f)$, the mapis well defined and depends only on the class $[\delta ] \in \pi _0(X_\beta )$.

#### Proof

Immediate by combining Proposition 5.9 and Lemma 5.12. $\square$

#### Definition 5.14

The *classical evaluation map attached to* $\chi$ *and the action* $\bullet$ is the map

$$\begin{aligned} \mathscr {E}\hspace{-1pt}{\scriptstyle \mathscr {V}}_{\chi }^{[\bullet ]} :=\sum _{\delta \in \pi _{0}(X_{\beta })} \mathscr {E}\hspace{-1pt}{\scriptstyle \mathscr {V}}_{\chi ,[\delta ]}^{[\bullet ]} : \textrm{H}^{t}_{\textrm{c}}(S_K,\mathscr {V}_\lambda ^\vee (L)) \longrightarrow L. \end{aligned}$$

It depends on the choices of $\kappa _{\textbf{j}}$ and a basis $u_{\textbf{j}}$ of the line $V_{\chi ,[p]}^H \cong L(\chi )$, each unique up to scalar.

As mentioned previously, there are two natural choices of action $\bullet$ on $V_\lambda ^\vee (L)$: the standard $\cdot$-action (which connects more cleanly to the theory from §4), and the $*$-action inherited as a quotient of $\mathcal {D}_\lambda$. We get two evaluation maps $\mathscr {E}\hspace{-1pt}{\scriptstyle \mathscr {V}}_{\chi }^{[\cdot ]}$ and $\mathscr {E}\hspace{-1pt}{\scriptstyle \mathscr {V}}_{\chi }^{[*]}$. Since the only dependence on $\bullet$ is in the map $\tau _\beta ^\bullet$, from (2.12) we have (recalling $\chi$ has conductor related to $p^\beta$)

5.11 $$\begin{aligned} \mathscr {E}\hspace{-1pt}{\scriptstyle \mathscr {V}}_{\chi }^{[*]} = \lambda (t_p^\beta ) \times \mathscr {E}\hspace{-1pt}{\scriptstyle \mathscr {V}}_\chi ^{[\cdot ]}. \end{aligned}$$

### Comparison between $\textrm{Ev}_\chi$nd $\mathscr {E}\hspace{-1pt}{\scriptstyle \mathscr {V}}_\chi ^{[\cdot ]}$

We now connect the evaluation with $\bullet = \cdot$ to Definition 5.5 and hence to §4. Let $\phi \in \textrm{H}^{t}_{\textrm{c}}(S_K,\mathcal {V}_\lambda ^{\vee }(L))$, and let $\upsilon$ denote the natural isomorphismfrom §2.6. Then, taking $\cdot$-actions everywhere:

#### Proposition 5.15

We have $$\textrm{Ev}_{\chi }(\phi ) = \mathscr {E}\hspace{-1pt}{\scriptstyle \mathscr {V}}_{\chi }^{[\cdot ]}(\upsilon (\phi )).$$

#### Proof

We have a commutative diagram (cf. [23, Prop. 4.6] and [5, Prop. 4.1])where the horizontal maps are induced by the stated maps of local systems. In particular,

$$\begin{aligned} \delta _p^{-1} \cdot \textrm{Ev}_{\beta ,\delta }(\phi ) = \mathscr {E}\hspace{-1pt}{\scriptstyle \mathscr {V}}_{\beta ,\delta }^{[\cdot ]}(\upsilon (\phi )) \end{aligned}$$

as elements of $(V_\lambda ^\vee (L))_{\Gamma _{\beta ,\delta }}$. Composing with the $H(\textbf{Q}_p)$-module map $\kappa _{\textbf{j}}$ gives

5.12 $$\begin{aligned} (w_p^{-\textbf{j}}\otimes w_p^{\underline{\textsf{w}}+\textbf{j}})(\delta _p)\Big [\kappa _{\textbf{j}}\circ \textrm{Ev}_{\beta ,\delta }(\phi )\Big ] = \Big [\kappa _{\textbf{j}}\circ \mathscr {E}\hspace{-1pt}{\scriptstyle \mathscr {V}}_{\beta ,\delta }^{[\cdot ]}(\upsilon (\phi ))\Big ]. \end{aligned}$$

Now consider both sides as elements of $V_{\chi ,[p]}^H$ by Lemma 5.12, and act by $\delta$ on both sides (via the $H(\textbf{A})$ action on $V_{\chi ,[p]}^H$). On the left-hand side, the factor $(w_p^{\textbf{j}}\otimes w_p^{-\underline{\textsf{w}}-\textbf{j}})(\delta _p)$ in $\chi _{[p]}\otimes \chi _{[p]}^{-1}\eta _{[p]}^{-1}$ cancels with the left-most term in (5.12), so we have

$$\begin{aligned} \delta \cdot \left( (w_p^{-\textbf{j}}\otimes w_p^{\underline{\textsf{w}}+\textbf{j}})(\delta _p) \Big [\kappa _{\textbf{j}}\circ \textrm{Ev}_{\beta ,\delta }(\phi )\Big ]\right) =(\chi \otimes \chi ^{-1}\eta ^{-1})(\delta ) \cdot \Big [ \kappa _{\textbf{j}} \circ \textrm{Ev}_{\beta ,\delta }(\phi )\Big ]. \end{aligned}$$

On the right-hand side we get, by definition, $\mathscr {E}\hspace{-1pt}{\scriptstyle \mathscr {V}}_{\chi ,[\delta ]}^{[\cdot ]}(\upsilon (\phi )).$ Summing both sides over $\delta \in \pi _0(X_\beta )$ completes the proof. $\square$

The following theorem, now using the $*$-action, summarises the last three sections. As in the last line of the proof of [23, Thm. 4.7]), the *global Gauss sum* is

5.13 $$\begin{aligned} \tau (\chi _f) :=\prod _{\chi _v \text { unramified}}\chi _v(\varpi _v)^{-\delta _v} \cdot \prod _{\chi _v \text { ramified}} \tau (\chi _v); \end{aligned}$$

this is different from some treatments, as our $\psi$ has conductor $\mathfrak {d}^{-1}$, rather than $\widehat{\mathcal {O}}_F$.

#### Theorem 5.16

Let $\pi$ and $\varphi _f^{\textrm{FJ},\alpha }$ be as in Test Data 3.12. For all $\chi \in \textrm{Crit}(\pi )$, we have

5.14 $$\begin{aligned} i_p^{-1}\circ \mathscr {E}\hspace{-1pt}{\scriptstyle \mathscr {V}}_{\chi }^{[*]}\circ \Theta _{[\omega ]}^{K,i_p}(\varphi _f^{\textrm{FJ},\alpha }) =&A \cdot \chi _{[p]}(\det w_n) \cdot \lambda (t_p^\beta ) \\&\times \tau (\chi _f)^n\cdot \prod _{\mathfrak {p}|p} Q'(\pi _{\mathfrak {p}},\chi _{\mathfrak {p}}) \cdot \frac{L^{(p)}\big (\pi \times \chi ,\tfrac{1}{2}\big )}{\Omega _{\pi ,\chi _\infty }},\nonumber \end{aligned}$$

where

$$\begin{aligned} Q'(\pi _{\mathfrak {p}},\chi _{\mathfrak {p}})=\left\{ \begin{array}{cl} q_{\mathfrak {p}}^{\beta _{\mathfrak {p}}\left( \tfrac{n^2-n}{2}\right) } & : \chi _{\mathfrak {p}} \text { ramified},\\ \displaystyle \alpha _{\mathfrak {p}} \cdot \prod _{i=n+1}^{2n} \frac{1-\theta _{\mathfrak {p},i}^{-1}\chi _{\mathfrak {p}}^{-1}(\varpi _{\mathfrak {p}})q_{\mathfrak {p}}^{-1/2}}{1-\theta _{\mathfrak {p},i}\chi _{\mathfrak {p}}(\varpi _{\mathfrak {p}})q_{\mathfrak {p}}^{-1/2}} & : \chi _{\mathfrak {p}} \text { unramified},\end{array}\right. \end{aligned}$$

and

5.15 $$\begin{aligned} A :=\frac{1}{\textrm{vol}(L_1)} \cdot \prod _{\mathfrak {p}|p} \left( \frac{q_{\mathfrak {p}}^n}{(q_{\mathfrak {p}}-1)^n} \cdot q_{\mathfrak {p}}^{\delta _{\mathfrak {p}}(n^2-n)/2}\right) \end{aligned}$$

is a constant independent of $\chi$. Here $L_1 :=L_{(1,...,1)}$ (i.e. we take $\beta _{\mathfrak {p}} = 1$ for all $\mathfrak {p}$), and we consider $\det (w_n)$ as an element of $(\mathcal {O}_F\otimes \textbf{Z}_p)^\times$.

#### Proof

By (5.11) (to pass from $*$ to $\cdot$ evaluations, introducing $\lambda (t_p^\beta )$), Corollary 4.5 and Lemma 5.6, noting that $u^{-1}t_p^\beta$ is trivial outside primes above *p*, we have

$$\begin{aligned} i_p^{-1}\circ \mathscr {E}\hspace{-1pt}{\scriptstyle \mathscr {V}}_{\chi }^{[*]}\circ \Theta _{[\omega ]}^{K,i_p}(\varphi _f^{\textrm{FJ},\alpha }) = \lambda (t_p^\beta )&\cdot \frac{ w^{\textbf{j}}(\det w_n)}{\textrm{vol}(L_\beta )} \cdot \prod _{v\not \mid p\infty }\chi _v(\varpi _v)^{-n\delta _v}\\&\times \prod _{\mathfrak {p}|p}Z_{\mathfrak {p}}\big ([u^{-1}t_{\mathfrak {p}}^{\beta _{\mathfrak {p}}}\cdot \varphi _{\mathfrak {p}}^{\alpha _{\mathfrak {p}}}],\chi _{\mathfrak {p}},\tfrac{1}{2}\big ) \cdot \frac{L^{(p)}\big (\pi \times \chi ,\tfrac{1}{2}\big )}{\Omega _{\pi ,\chi _\infty }}. \end{aligned}$$

By [13, Lem. 4.4] (cf. Lemma 5.2), we have

5.16 $$\begin{aligned} \textstyle \textrm{vol}(L_\beta ) = \textrm{vol}(L_1) \cdot \delta _B(t_p^\beta ) = \textrm{vol}(L_1) \cdot \prod _{\mathfrak {p}|p}q_{\mathfrak {p}}^{-\beta _{\mathfrak {p}}n^2}. \end{aligned}$$

The twisted local zeta integrals at *p* were evaluated^2^ in Proposition 3.6. In the ramified case, the term $q_{\mathfrak {p}}^{-\beta _{\mathfrak {p}}n^2}$ is the difference between *Q* (from that proposition) and $Q'$ (here); in the unramified case, it cancels with the power of $q_{\mathfrak {p}}^{-n^2}$ that appears in *Q*. Via §2.5, the local terms involving $\det w_n$ combine to give $\chi _{[p]}(\det w_n)$. Combining all of this, we get the factor $Q'(\pi _{\mathfrak {p}},\alpha _{\mathfrak {p}})$ and $\chi _{\mathfrak {p}}(\varpi _{\mathfrak {p}}^{-\delta _{\mathfrak {p}}})$ (resp. $\tau (\chi _{\mathfrak {p}})$) if $\chi _{\mathfrak {p}}$ is unramified (resp. ramified). Finally we conclude by the identity (5.13). $\square$

## *p*-adic interpolation of evaluation maps

We will interpolate the *L*-values appearing on the right-hand side of Theorem 5.16 by interpolating the left-hand side, i.e. $\mathscr {E}\hspace{-1pt}{\scriptstyle \mathscr {V}}_{\chi }^{[*]}$, as $\chi$ varies. The main result of §6 is Proposition 6.12.

### Alignment of branching laws

The evaluation maps of the previous section depended on choices of bases

6.1 $$\begin{aligned} \kappa _{\textbf{j}} \in {{\,\textrm{Hom}\,}}_{H(\textbf{Q}_p)}(V_\lambda ^\vee (L), V^H_{\textbf{j},-\underline{\textsf{w}}-\textbf{j}}(L)), \qquad u_{\textbf{j}} \in V^H_{\textbf{j},-\underline{\textsf{w}}-\textbf{j}}(L) \cong L. \end{aligned}$$

The choices can be combined into a single choice $\kappa _{\textbf{j}}^\circ : V_\lambda ^\vee (L) \rightarrow L$ defined by

6.2 $$\begin{aligned} \kappa _{\textbf{j}}(\mu ) = \kappa _{\textbf{j}}^\circ (\mu ) \cdot u_{\textbf{j}} \qquad \forall \mu \in V_{\lambda }^\vee (L). \end{aligned}$$

In Theorem 5.16, these choices manifested themselves on the left-hand side in the definition of $\mathscr {E}\hspace{-1pt}{\scriptstyle \mathscr {V}}_{\chi }^{[*]}$, and on the right-hand side (via $i_p$) in the zeta integral at infinity. To interpolate the $\mathscr {E}\hspace{-1pt}{\scriptstyle \mathscr {V}}_{\chi }^{[*]}$ requires a careful alignment of the choices as $\textbf{j}$ varies, which we carry out here. The main idea is that we can collapse all the different choices (as $\textbf{j}$ varies) of branching law $\kappa _{\textbf{j}}$ for $H \subset G$ onto a *single* choice of branching law for $G_n :=\textrm{Res}_{F/Q}({{\,\textrm{GL}\,}}_n) \subset H$, diagonally embedded.

Write $\lambda = (\lambda ',\lambda '')$, where $\lambda ',\lambda ''$ are two weights for $G_n$. Then as *H*-representations $V_\lambda ^H \cong V_{\lambda '}^{G_n} \otimes V_{\lambda ''}^{G_n}$. We can restrict this under the diagonal embedding of $G_n$, obtaining:

#### Lemma 6.1

The restriction $V_{\lambda }^H(L)|_{G_n}$ to a diagonal copy of $G_n$ contains the $G_n$-representation $w_p^{\underline{\textsf{w}}}\circ \det$ with multiplicity 1.

#### Proof

For each $\sigma \in \Sigma$, the individual weight $\lambda _\sigma \in \textbf{Z}^{2n}$ is pure by §2.3; that is, we have $\lambda _{\sigma ,i} + \lambda _{\sigma ,2n+1-i} = \textsf{w}_\sigma$ for $1 \leqslant i \leqslant n$. Combining over all *i*, this means that

$$\begin{aligned} \lambda ''_\sigma = (\lambda _{\sigma ,n+1},\dots , \lambda _{\sigma ,2n}) = (-\lambda _{\sigma ,n} + \textsf{w}_\sigma , \dots , -\lambda _{\sigma ,1} + \textsf{w}_\sigma ) = (\lambda '_\sigma )^\vee + (\textsf{w}_\sigma ,...,\textsf{w}_\sigma ), \end{aligned}$$

noting that $(\lambda _\sigma ')^\vee _i = -\lambda _{\sigma , n+1-i}$. Thus $\lambda '' = (\lambda ')^\vee + (\underline{\textsf{w}},\cdots , \underline{\textsf{w}})$. It follows that

$$\begin{aligned} V_{\lambda '}^{G_n} \otimes V_{\lambda ''}^{G_n} \cong V_{\lambda '}^{G_n} \otimes (V_{\lambda '}^{G_n})^\vee \otimes \textrm{det}^{\underline{\textsf{w}}}, \end{aligned}$$

which contains $\det ^{\underline{\textsf{w}}}$ with multiplicity 1. $\square$

Fix a generator $v^H_\lambda \in w_p^{\underline{\textsf{w}}}\circ \det \subset V_{\lambda }^H(L)|_{G_n}$. Recall the description $V_\lambda = {{\,\textrm{Ind}\,}}_{\overline{Q}}^G V_\lambda ^H$ from (2.7). Let

6.3 $$\begin{aligned} N_Q^\times (\textbf{Z}_p) :=\left\{ \left( {\begin{smallmatrix}1 &  X\\ 0 &  1\end{smallmatrix}}\right) \in N_Q(\textbf{Z}_p) : X \in G_n(\textbf{Z}_p)\right\} . \end{aligned}$$

#### Proposition 6.2

(i) For each $\textbf{j}$ critical for $\lambda$, there exists a unique
  $$\begin{aligned} \big [v_{\textbf{j}} : G(\textbf{Z}_p) \rightarrow V_\lambda ^H(L) \big ] \in V_\lambda (L) \end{aligned}$$
  with
  $$\begin{aligned} v_{\textbf{j}}\left[ \left( {\begin{smallmatrix}1 &  X\\ 0 &  1\end{smallmatrix}}\right) \right] = w_p^{\textbf{j}}(\det (w_nX)) \cdot \Big (\left\langle \left( {\begin{smallmatrix}X &  \\  &  1\end{smallmatrix}}\right) \right\rangle _\lambda \cdot v_\lambda ^H\Big ) \end{aligned}$$
  for all $$\left( {\begin{smallmatrix}1 &  X\\ 0 &  1\end{smallmatrix}}\right) \in N_Q^\times (\textbf{Z}_p)$$.
(ii) $$v_{\textbf{j}}$$ generates $$V^H_{-\textbf{j},\underline{\textsf{w}}+\textbf{j}}(L) \subset V_\lambda (L)|_{H(\textbf{Q}_p)}.$$

#### Proof

This follows [12, Lem. 5.11]. Take *some* choices of $\kappa _{\textbf{j}}$ and $u_{\textbf{j}}$. These define dual bases

6.4 $$\begin{aligned} \kappa _{\textbf{j}}^\vee \in {{\,\textrm{Hom}\,}}_{H(\textbf{Q}_p)}(V_{-\textbf{j},\underline{\textsf{w}}+\textbf{j}}^H(L), V_\lambda (L)), \qquad u_{\textbf{j}}^\vee \in V^H_{-\textbf{j},\underline{\textsf{w}}+\textbf{j}}(L). \end{aligned}$$

Thus $\kappa _{\textbf{j}}^\vee (u_{\textbf{j}}^\vee ) \in V_\lambda (L)$; and via (2.7), we may define

$$\begin{aligned} v_{\lambda ,\textbf{j}}^H :=\kappa _{\textbf{j}}^\vee (u_{\textbf{j}}^\vee )\left[ \left( {\begin{smallmatrix}1_n &  1_n\\ 0 &  1_n\end{smallmatrix}}\right) \right] \in V_\lambda ^H(L). \end{aligned}$$

Arguing exactly as in [12, Lem. 5.8], we see $v_{\lambda ,\textbf{j}}^H$ is a nonzero element of $w_p^{\underline{\textsf{w}}}\circ \det \subset V_{\lambda }^H(L)|_{G_n}$, and hence—up to rescaling $\kappa _{\textbf{j}}$—we may assume $v_{\lambda ,\textbf{j}}^H = v_{\lambda }^H$, independent of $\textbf{j}$.

For these rescaled choices, define $v_{\textbf{j}} :=\kappa _{\textbf{j}}^\vee (u_{\textbf{j}}^\vee )$. Then (i) follows exactly as in [12, Lem. 5.7], with uniqueness following from Zariski-density of $\overline{N}_QHN_Q^\times (\textbf{Z}_p) \subset G(\textbf{Z}_p)$ (as in [12, Lem. 5.11(i)]). Part (ii) is then identical to [12, Lem. 5.11(ii)]. $\square$

#### Definition 6.3

We fix the choice of $\kappa _{\textbf{j}}^\circ : V_{\lambda }^\vee (L) \rightarrow L$ by setting $\kappa _{\textbf{j}}^\circ (\mu ) = \mu (v_{\textbf{j}})$. Note this corresponds to the choices of $\kappa _{\textbf{j}}$ and $u_{\textbf{j}}$ in the proof, after rescaling so that the attached $v_{\lambda ,\textbf{j}}^H = v_{\lambda }^H$.

### Branching laws for distributions

We now construct a ‘master branching law’ $\kappa _\lambda$, interpolating all the $\kappa _{\textbf{j}}$ above. Let $\mathcal {A}({{\,\textrm{Gal}\,}}_p,L)$ be the space of locally analytic functions on ${{\,\textrm{Gal}\,}}_p$, with an $H(\textbf{A})$-action by

$$\begin{aligned} (h_1,h_2) * f(x) :=\eta _{[p]}(h_2) f(\det (h_1^{-1}h_2) x). \end{aligned}$$

This induces a dual left action on $\mathcal {D}({{\,\textrm{Gal}\,}}_p,L) :=\textrm{Hom}_{\textrm{cts}}(\mathcal {A}({{\,\textrm{Gal}\,}}_p,L),L)$. Recall if $\chi \in \textrm{Crit}_p(\pi )$, then $\chi _{[p]}$ induces a character on ${{\,\textrm{Gal}\,}}_p$ via §2.5. The following is immediate.

#### Lemma 6.4

(i) $H(\textbf{R})^\circ$ and $H(\textbf{Q})$ act trivially on $\mathcal {D}({{\,\textrm{Gal}\,}}_p,L)$.
(ii) The map $\mu \mapsto \mu (\chi _{[p]})$ defines an $H(\textbf{A})$-module map $\mathcal {D}({{\,\textrm{Gal}\,}}_p,L) \rightarrow V_{\chi ,[p]}^H(L)$.

Recall from (2.4) there is a natural map $(\mathcal {O}_F\otimes \textbf{Z}_p)^\times \rightarrow {{\,\textrm{Gal}\,}}_p$, which we denote $\jmath$. Given $f \in \mathcal {A}({{\,\textrm{Gal}\,}}_p,L)$ and $x \in (\mathcal {O}_F\otimes \textbf{Z}_p)^\times$, abusing notation we write $f(x) :=f(\jmath (x))$. For such *f*, define a map

6.5 $$\begin{aligned} v_\lambda (f) : N_Q^\times (\textbf{Z}_p)&\longrightarrow V_\lambda ^H(L),\\ \left( {\begin{smallmatrix}1 &  X\\ 0 &  1\end{smallmatrix}}\right)&\longmapsto f(\det (w_n X)) \left( \langle \left( {\begin{smallmatrix}X &  \\  &  1\end{smallmatrix}}\right) \rangle _\lambda \cdot v_\lambda ^H\right) ,\nonumber \end{aligned}$$

noting $\det (X) \in (\mathcal {O}_F\otimes \textbf{Z}_p)^\times$ by the definition (6.3) of $N_Q^\times (\textbf{Z}_p)$. Extending by 0, $v_\lambda (f)$ is a locally analytic function $N_Q(\textbf{Z}_p) \rightarrow V_\lambda ^H(L)$, whence it defines an element $v_\lambda (f) \in \mathcal {A}_\lambda$ via the parahoric transformation law (2.9).

If there was a function $w_p^{\textbf{j}} \in \mathcal {A}({{\,\textrm{Gal}\,}}_p,L)$, then comparing Proposition 6.2 with (6.5), we would formally have $v_\lambda (w_p^{\textbf{j}})(n) = v_{\textbf{j}}(n)$ for $n \in N_Q^\times (\textbf{Z}_p)$. Unfortunately the function $w_p^{\textbf{j}}$ on $(F\otimes \textbf{Q}_p)^\times$ does *not* induce a function on ${{\,\textrm{Gal}\,}}_p$ in general (since it is not $\textbf{Q}^\times$-invariant). However, if $\chi \in \textrm{Crit}_p(\pi )$ with infinity type $\textbf{j}$ and conductor dividing $p^\beta$, then $\chi _{[p]}$ *is* a function on ${{\,\textrm{Gal}\,}}_p$, and $\chi _{[p]}(x) = w_p^{\textbf{j}}(x)$ for $x \in \textbf{A}_F^\times$ with $x \equiv 1\hspace{2pt}(\textrm{mod}\hspace{2pt}p^\beta )$. As such, recalling that $\beta = (\beta _{\mathfrak {p}}) \in \textbf{Z}_{\geqslant 1}^{\mathfrak {p}|p}$, let:

- $$\mathscr {U}_\beta = \jmath (1+p^\beta \mathcal {O}_F\otimes \textbf{Z}_p) \subset {{\,\textrm{Gal}\,}}_p$$,
- $\mathcal {A}(\mathscr {U}_\beta ,L) \subset \mathcal {A}({{\,\textrm{Gal}\,}}_p,L)$ be the space of functions supported on $\mathscr {U}_\beta$,
- $$N^\beta _Q(\textbf{Z}_p) :=\{n \in N_Q(\textbf{Z}_p) : n \equiv \left( {\begin{smallmatrix}1 &  w_n\\ 0 &  1_n\end{smallmatrix}}\right) \hspace{2pt}(\textrm{mod}\hspace{2pt}p^\beta )\} \subset N_Q^\times (\textbf{Z}_p),$$
- $$J_p^\beta :=J_p \cap \overline{N}_Q(\textbf{Z}_p)H(\textbf{Z}_p)N_Q^\beta (\textbf{Z}_p) \subset J_p$$,
- $\mathcal {A}_\lambda ^\beta \subset \mathcal {A}_\lambda$ (resp. $\mathcal {D}_\lambda ^\beta \subset \mathcal {D}_\lambda$) be the space of functions (resp. distributions) supported on $J_p^\beta$.

If $$n = \left( {\begin{smallmatrix}1 &  X\\ 0 &  1\end{smallmatrix}}\right) \in N_Q^\beta (\textbf{Z}_p)$$, then $X \equiv w_n \hspace{2pt}(\textrm{mod}\hspace{2pt}p^\beta )$, so $\det (w_nX) \equiv 1 \hspace{2pt}(\textrm{mod}\hspace{2pt}p^\beta )$. If $f \in \mathcal {A}(\mathscr {U}_\beta ,L)$, we can thus define a function $v_\lambda ^\beta (f) : \textbf{N}_Q^\beta (\textbf{Z}_p) \rightarrow V_\lambda ^H(L)$ exactly as in (6.5). After extending by 0 to $N_Q(\textbf{Z}_p)$, as above we obtain a function

$$\begin{aligned} v_\lambda ^\beta : \mathcal {A}(\mathscr {U}_\beta ,L) \longrightarrow \mathcal {A}_\lambda ^\beta . \end{aligned}$$

Then, recalling that $\chi$ has conductor dividing $p^\beta$ and infinity type $\textbf{j}$:

#### Lemma 6.5

If $$g \in J_p^\beta $$, then $$v_\lambda ^\beta (\chi _{[p]})(g) = v_{\textbf{j}}(g)$$.

#### Proof

As both $v_\lambda ^\beta (\chi _{[p]})$ and $v_{\textbf{j}}$ are functions $J_p \rightarrow V_\lambda ^H(L)$ satisfying the same parahoric transformation law, it suffices to show this for $$g = \left( {\begin{smallmatrix}1 &  X\\ 0 &  1\end{smallmatrix}}\right) \in N_Q^\beta (\textbf{Z}_p)$$. Then $\det (w_nX) \equiv 1\hspace{2pt}(\textrm{mod}\hspace{2pt}p^\beta )$, so $\chi _{[p]}(\det (w_nX)) = w_p^{\textbf{j}}(\det (w_nX))$. Thus when we plug $\chi _{[p]}$ into (6.5), we recover the formula for $v_{\textbf{j}}$ in Proposition 6.2. $\square$

Dualising $v_\lambda ^\beta$ gives a map $\kappa _\lambda ^\beta : \mathcal {D}_\lambda ^\beta \rightarrow \mathcal {D}(\mathscr {U}_\beta ,L) \subset \mathcal {D}({{\,\textrm{Gal}\,}}_p,L)$.

#### Proposition 6.6

If $\chi \in \textrm{Crit}_p(\pi )$ has conductor dividing $p^\beta$ and infinity type $\textbf{j}$, we have a commutative diagram

#### Proof

Let $$\mu \in \mathcal {D}_\lambda ^\beta $$. Then

$$\begin{aligned} \kappa _\lambda ^\beta (\mu )(\chi _{[p]}) = \int _{{{\,\textrm{Gal}\,}}_p} \chi _{[p]} \cdot \textrm{d}\kappa _\lambda ^\beta (\mu ) = \int _{{{\,\textrm{Gal}\,}}_p}v_\lambda ^\beta (\chi _{[p]}) \cdot \textrm{d}\mu = \int _{J_p^\beta }v_\lambda ^\beta (\chi _{[p]}) \cdot \textrm{d}\mu = \int _{J_p^\beta }v_{\textbf{j}} \cdot \textrm{d}\mu = \kappa _{\textbf{j}}^\circ \circ r_\lambda (\mu ), \end{aligned}$$

where we have used that $\mu$ has support on $J_p^\beta$, and $\mu (v_{\textbf{j}}) = \mu (r_\lambda (v_{\textbf{j}}))$ since $v_{\textbf{j}} \in V_\lambda$. $\square$

### Overconvergent evaluation maps

Recall *K* (hence $L_\beta$) acts on $\mathcal {D}_\lambda$ via §2.7.3 by projection to $K_p \subset J_p$, giving a local system $\mathscr {D}_\lambda$ on $S_K$ by §2.6.

#### Lemma 6.7

The action of $L_\beta \subset K$ preserves $\mathcal {D}_\lambda ^\beta$.

#### Proof

If $f \in \mathcal {A}_\lambda ^\beta$, and $\ell = (\ell _1,\ell _2) \in L_\beta$, then for any $$\left( {\begin{smallmatrix}1 &  X\\ 0 &  1\end{smallmatrix}}\right) \in N_Q(\textbf{Z}_p)$$, we have $$(\ell * f)\left( {\begin{smallmatrix}1 &  X\\ 0 &  1\end{smallmatrix}}\right) = \langle \ell \rangle _H \cdot f\left( {\begin{smallmatrix}1 &  \ell _1^{-1}X\ell _2\\ 0 &  1\end{smallmatrix}}\right) $$. By the proof of [13, Lem. 4.5], we have $\ell _2 \equiv w_n\ell _1w_n \hspace{2pt}(\textrm{mod}\hspace{2pt}p^\beta )$; so $\ell _1^{-1}X\ell _2 \equiv w_n \hspace{2pt}(\textrm{mod}\hspace{2pt}p^\beta )$ if and only if $X \equiv \ell _2w_n\ell _2^{-1} \equiv w_n \hspace{2pt}(\textrm{mod}\hspace{2pt}p^\beta )$. Thus $\ell * f$ has support on $N_Q^\beta (\textbf{Z}_p)$ if *f* does; so $L_\beta$ preserves $\mathcal {A}_\lambda ^\beta$, hence $\mathcal {D}_\lambda ^\beta$. $\square$

To make Proposition 6.6 useful, we need the following support condition.

#### Lemma 6.8

The map

$$\begin{aligned} \mathscr {E}\hspace{-1pt}{\scriptstyle \mathscr {V}}_{\beta ,\delta }^{\mathcal {D}_\lambda ,[*]} : \textrm{H}^{t}_{\textrm{c}}(S_K,\mathscr {D}_\lambda ) \rightarrow (\mathcal {D}_\lambda )_{\Gamma _{\beta ,\delta }} \end{aligned}$$

has image in $(\mathcal {D}_\lambda ^\beta )_{\Gamma _{\beta ,\delta }} \subset (\mathcal {D}_\lambda )_{\Gamma _{\beta ,\delta }}$.

#### Proof

Identical to [13, Lem. 12.4]. $\square$

The following, and Lemma 6.4, allows us to use Proposition 5.9.

#### Lemma 6.9

The map $\kappa _\lambda ^\beta : \mathcal {D}_\lambda ^\beta \rightarrow \mathcal {D}({{\,\textrm{Gal}\,}}_p,L)$ is a map of $L_\beta$-modules.

#### Proof

It suffices to prove that $v_\lambda ^\beta : \mathcal {A}(\mathscr {U}_\beta ,L) \rightarrow \mathcal {A}_\lambda ^\beta$ is a map of $L_\beta$-modules. This is almost identical to [12, Prop. 6.11(ii)]. We observe that if $f \in \mathcal {A}({{\,\textrm{Gal}\,}}_p,L)$ and $\ell = (\ell _1,\ell _2) \in L_\beta$, then $(\ell * f)(x) = w_p(\det (\ell _{2,p}))^{\underline{\textsf{w}}}f(\det (\ell _{1,p}^{-1}\ell _{2,p}))$, since $\eta (\det (\ell _2)) = 1$ (as in Lemma 5.4) and the prime-to-*p* part of $\ell _1^{-1}\ell _2$ lies in $\mathscr {U}(p^\infty )$, i.e. acts trivially on $\textrm{Cl}_F^+(p^\infty )$. The rest of the proof proceeds exactly as *op. cit*. $\square$

#### Proposition 6.10

For any $\delta \in H(\textbf{A}_f)$, the map

$$\begin{aligned} \mathscr {E}\hspace{-1pt}{\scriptstyle \mathscr {V}}_{\beta ,[\delta ]}^{\dagger } : \textrm{H}^{t}_{\textrm{c}}(S_K,\mathscr {D}_\lambda ^\vee (L)) \xrightarrow {\ \mathscr {E}\hspace{-1pt}{\scriptstyle \mathscr {V}}_{\beta ,\delta }^{\mathcal {D}_\lambda ,[*]} \ } \big (\mathcal {D}_{\lambda }^\beta (L)\big )_{\Gamma _{\beta ,\delta }} \xrightarrow {\kappa _{\lambda }^\beta } \mathcal {D}({{\,\textrm{Gal}\,}}_p,L) \xrightarrow {\ \cdot \delta \ } \mathcal {D}({{\,\textrm{Gal}\,}}_p,L) \end{aligned}$$

is well defined and depends only on the class $[\delta ] \in \pi _0(X_\beta )$.

#### Proof

Immediate by combining Proposition 5.9 with Lemmas 6.4(i) and 6.9. $\square$

#### Definition 6.11

The *overconvergent evaluation map of level* $\beta$ is the map

$$\begin{aligned} \mathscr {E}\hspace{-1pt}{\scriptstyle \mathscr {V}}_{\beta }^{\dagger } :=\sum _{\delta \in \pi _{0}(X_{\beta })} \mathscr {E}\hspace{-1pt}{\scriptstyle \mathscr {V}}_{\beta ,[\delta ]}^{\dagger } : \textrm{H}^{t}_{\textrm{c}}(S_{K},\mathscr {D}_{{\lambda }^{\vee }}(L)) \longrightarrow \mathcal {D}({{\,\textrm{Gal}\,}}_p,L). \end{aligned}$$

### Interpolation of classical evaluation maps

The map $r_\lambda$ from (2.10) is by definition a $\Delta _p$-module map when $V_\lambda ^\vee$ is given the $*$-action, hence induces a $U_p^*$-equivariant map

6.6 $$\begin{aligned} r_\lambda : \textrm{H}^{t}_{\textrm{c}}(S_K,\mathscr {D}_\lambda ) \longrightarrow \textrm{H}^{t}_{\textrm{c}}(S_K,\mathscr {V}_\lambda ^\vee ). \end{aligned}$$

#### Proposition 6.12

For any $\chi$ of conductor dividing $p^\beta$, we have a commutative diagram

#### Proof

It suffices to check every square incommutes, since the top row is $\mathscr {E}\hspace{-1pt}{\scriptstyle \mathscr {V}}^{\dagger }_{\beta }$ and the bottom row $\mathscr {E}_{\chi }^{[*]}$. The left-most square commutes by Lemma 5.8 (noting the top arrow is well defined by Lemma 6.8). The middle square commutes by Proposition 6.6. The right-most square commutes by Lemma 6.4(ii), noting that $\delta$ acts on the bottom row via the identification $L \cong V_{\chi ,[p]}^H(L)$. $\square$

## The *p*-adic *L*-function

### Construction

Let now $\pi$ be a RASCAR of weight $\lambda$, spherical at all $\mathfrak {p}|p$, and let $\tilde{\pi }= (\pi , \{\alpha _{\mathfrak {p}}\}_{\mathfrak {p}}\}$ be a regular spin *Q*-refinement as in §3.2. Let $\varphi _{f}^{\textrm{FJ},\alpha } \in \pi _f$ and $[\omega ]$ be chosen as in Test Data 3.12, and let

7.1 $$\begin{aligned} \phi _{\tilde{\pi }} :=\Theta _{[\omega ]}^{K,i_p}(\varphi _f^{\textrm{FJ},\alpha }) \in \textrm{H}^{t}_{\textrm{c}}(S_K,\mathscr {V}_\lambda ^\vee (\overline{\textbf{Q}}_p)). \end{aligned}$$

There exists some finite extension $L/\textbf{Q}_p$ such that $\phi _{\tilde{\pi }}$ is defined over *L*, rather than $\overline{\textbf{Q}}_p$. The map $\Theta _{[\omega ]}^{K,i_p}$ is Hecke-equivariant when we give the right-hand side the $\cdot$-action, hence

$$\begin{aligned} U_{\mathfrak {p}}^\cdot \phi _{\tilde{\pi }} = \alpha _{\mathfrak {p}}\phi _{\tilde{\pi }}, \qquad U_{\mathfrak {p}}^*\phi _{\tilde{\pi }} = \lambda (t_{\mathfrak {p}}) \alpha _{\mathfrak {p}} \phi _{\tilde{\pi }}, \end{aligned}$$

the second via (2.12). Let $\alpha _{\mathfrak {p}}^\circ :=\lambda (t_{\mathfrak {p}})\alpha _{\mathfrak {p}}$, the $U_{\mathfrak {p}}^*$-eigenvalue.

#### Definition 7.1

Recall the map $r_\lambda$ from (6.6). We say $\tilde{\pi }$ is *non*-*Q*-*critical* if $r_{\lambda }$ becomes an isomorphism after restricting to the $\tilde{\pi }$-generalised eigenspaces for the Hecke algebra (precisely, the algebra $\mathcal {H}$ from [12, Def. 2.10]). We say $\tilde{\pi }$ has *non*-*Q*-*critical slope* if

$$\begin{aligned} e_{\mathfrak {p}} \cdot v_p(\alpha _{\mathfrak {p}}^\circ ) < \textrm{min}_{\sigma \in \Sigma (\mathfrak {p})}(1 + \lambda _{\sigma ,n} - \lambda _{\sigma ,n+1}) \qquad \text { for all }\,\,\mathfrak {p}|p, \end{aligned}$$

where $\Sigma (\mathfrak {p})$ is the set of  that extend to $F_{\mathfrak {p}} \hookrightarrow \overline{\textbf{Q}}_p$, and $e_{\mathfrak {p}}$ is the ramification index of $\mathfrak {p}|p$. (If $\tilde{\pi }$ is *ordinary* – i.e. $v_p(\alpha _{\mathfrak {p}}^\circ ) = 0$ for all $\mathfrak {p}$ – then it has non-*Q*-critical slope.)

The following is [10, Thm. 4.4] (see also [12, Thm. 3.17]).

#### Theorem 7.2

If $\tilde{\pi }$ has non-*Q*-critical slope, then it is non-*Q*-critical.

If $\tilde{\pi }$ is non-*Q*-critical, then there exists a unique Hecke eigenclass $\Phi _{\tilde{\pi }} \in \textrm{H}^{t}_{\textrm{c}}(S_K,\mathscr {D}_\lambda )$ with $r_\lambda (\Phi _{\tilde{\pi }}) = \phi _{\tilde{\pi }}$. If $\beta \in \textbf{Z}_{\geqslant 1}^{\mathfrak {p}|p}$, then let $(\alpha _p^\circ )^\beta = \prod _{\mathfrak {p}|p}(\alpha _{\mathfrak {p}}^\circ )^{\beta _{\mathfrak {p}}}$.

#### Definition 7.3

Let $\beta \in \textbf{Z}_{\geqslant 1}^{\mathfrak {p}|p}$. The *p*-*adic* *L*-*function attached to* $\tilde{\pi }$ *and* $i_p$ is

$$\begin{aligned} L_p^{i_p}(\tilde{\pi }) :=(\alpha _{p}^\circ )^{-\beta }\mathscr {E}\hspace{-1pt}{\scriptstyle \mathscr {V}}_{\beta }^{\dagger }(\Phi _{\tilde{\pi }}) \in \mathcal {D}({{\,\textrm{Gal}\,}}_p,L). \end{aligned}$$

By Proposition 5.10 (with $\bullet = *$), this is independent of $\beta$. Dependence on $i_p$ is (7.1) (via (5.1)).

### Growth conditions

The following is an adaptation of [35, Def. 2.14] for our setting. For $\textbf{m}= (m_{\mathfrak {p}}) \in \textbf{Z}_{\geqslant 1}^{\mathfrak {p}|p}$, let $p^{\textbf{m}} = \prod \mathfrak {p}^{m_{\mathfrak {p}}}$. We have an exact sequence

$$\begin{aligned} 0 \rightarrow \mathscr {U}_{\textbf{m}} \rightarrow {{\,\textrm{Gal}\,}}_p\rightarrow \textrm{Cl}_F^+(p^{\textbf{m}}) \rightarrow 0, \end{aligned}$$

where $\mathscr {U}_{\textbf{m}}$ is the image of $1+p^{\textbf{m}}\mathcal {O}_F\otimes \textbf{Z}_p$ under the natural map $(\mathcal {O}_F\otimes \textbf{Z}_p)^\times \rightarrow {{\,\textrm{Gal}\,}}_p$ from (2.4). Let

$$\begin{aligned} \mathcal {A}_{\textbf{m}}({{\,\textrm{Gal}\,}}_p,L) :=\{f \in \mathcal {A}({{\,\textrm{Gal}\,}}_p,L) : f\text { is analytic on every translate of }\,\, \mathscr {U}_{\textbf{m}}\}. \end{aligned}$$

Then $\mathcal {A}({{\,\textrm{Gal}\,}}_p,L) = \varinjlim _{\textbf{m}} \mathcal {A}_{\textbf{m}}({{\,\textrm{Gal}\,}}_p,L)$ is the union of all the $\mathcal {A}_{\textbf{m}}$’s. Moreover, each $\mathcal {A}_{\textbf{m}}$ is a Banach *L*-space with respect to a discretely valued norm $||\cdot ||_{\textbf{m}}$. Dualising gives a family of norms

7.2 $$\begin{aligned} ||\mu ||_{\textbf{m}}&:=\textrm{sup}_{f\in \mathcal {A}_{\textbf{m}}({{\,\textrm{Gal}\,}}_p,L)}\tfrac{|\mu (f)|}{||f||_{\textbf{m}}}\nonumber \\&= \textrm{sup}_{||f||_{\textbf{m}} \leqslant 1}|\mu (f)| \end{aligned}$$

on $\mathcal {D}({{\,\textrm{Gal}\,}}_p,L)$, which thus obtains the structure of a Fréchet module.

#### Definition 7.4

Let $\textbf{h}= (h_{\mathfrak {p}}) \in \textbf{Q}_{\geqslant 0}^{\mathfrak {p}|p}$. We say $\mu \in \mathcal {D}({{\,\textrm{Gal}\,}}_p,L)$ is *admissible of growth* $\textbf{h}$ if there exists $C \geqslant 0$ such that for each $\textbf{m}\in \textbf{Z}_{\geqslant 1}^{\mathfrak {p}|p},$ we have $||\mu ||_{\textbf{m}} \leqslant p^{\textbf{h}\textbf{m}}C,$ where $\textbf{h}\textbf{m}= (h_{\mathfrak {p}}m_{\mathfrak {p}})_{\mathfrak {p}}$.

Note that if $\mu$ is admissible of growth $(0)_{\mathfrak {p}|p}$, then it defines a bounded measure. Indeed, if $X \subset {{\,\textrm{Gal}\,}}_p$ is open compact, define its volume to be $\mu (1_X)$, where $1_X$ is the indicator function. For $\textbf{m}$, such that $1_X \in \mathcal {A}_{\textbf{m}}({{\,\textrm{Gal}\,}}_p,L)$, we see $|\mu (1_X)| = \tfrac{|\mu (1_X)|}{||1_X||_{\textbf{m}}} \leqslant ||\mu ||_{\textbf{m}} \leqslant C$, so $\mu$ is bounded.

#### Proposition 7.5

Let $\Phi \in \textrm{H}^{t}_{\textrm{c}}(S_K,\mathscr {D}_\lambda )$ be a Hecke eigenclass with $U_{\mathfrak {p}}^* \Phi = \alpha _{\mathfrak {p}}^\circ \Phi$ for all $\mathfrak {p}$. The distribution $\mathscr {E}\hspace{-1pt}{\scriptstyle \mathscr {V}}_{\beta }^{\dagger }(\Phi ) \in \mathcal {D}({{\,\textrm{Gal}\,}}_p,L)$ is admissible of growth $\textbf{h}_p :=(v_p(\alpha _{\mathfrak {p}}^\circ ))_{\mathfrak {p}|p}$.

#### Proof

This is a higher-variable version of [12, Prop. 6.20] and [5, Prop. 3.11], and it is proved essentially identically. We indicate changes of notation. One defines $\beta _{\textbf{m}} = (m_{\mathfrak {p}}e_{\mathfrak {p}})_{\mathfrak {p}|p}$ and replaces all instances of *m* *op. cit*. (a positive integer) with $\textbf{m}$. After (6.21) *op. cit*., *g* should instead be analytic on $N_Q^\beta (\textbf{Z}_p)$, and $\xi$ there replaced with $u^{-1}$ here. The space ${{\,\textrm{Gal}\,}}_p[\textrm{pr}_{\beta _m}([\delta ])]$ there is the translate of $\mathscr {U}_{\textbf{m}}$ by $\delta$ here. The rest of the proof is identical. $\square$

### Proof of Theorem A

Finally we assemble our results and prove Theorem A from the introduction. We have constructed the distribution $L_p^{i_p}(\tilde{\pi })$ in Definition 7.3. It has the required growth property by Proposition 7.5. For the interpolation, if $\chi$ has conductor dividing $p^\beta$, we have

$$\begin{aligned} \int _{{{\,\textrm{Gal}\,}}_p} \chi _{[p]} \cdot \textrm{d}L_p^{i_p}(\tilde{\pi }) = (\alpha _p^\circ )^{-\beta }\int _{{{\,\textrm{Gal}\,}}_p} \chi _{[p]} \cdot \textrm{d}\mathscr {E}\hspace{-1pt}{\scriptstyle \mathscr {V}}_{\beta }^{\dagger }(\Phi _{\tilde{\pi }}) = (\alpha _p^\circ )^{-\beta } \cdot \mathscr {E}\hspace{-1pt}{\scriptstyle \mathscr {V}}_{\chi }^{[*]}(r_\lambda (\Phi _{\tilde{\pi }})), \end{aligned}$$

by Proposition 6.12. Since $r_\lambda (\Phi _{\tilde{\pi }}) = \phi _{\tilde{\pi }} = \Theta _{[\omega ]}^{K,i_p}(\varphi _{f}^{\textrm{FJ},\alpha })$, this gives $(\alpha _p^\circ )^{-\beta }$ times the right-hand side of Theorem 5.16, which has a factor $\lambda (t_p^{\beta })$. By definition of $\alpha _{\mathfrak {p}}^\circ$, we have

$$\begin{aligned} \lambda (t_p^\beta )(\alpha _p^\circ )^{-\beta } = \alpha _p^{-\beta } = \prod _{\mathfrak {p}|p} \alpha _{\mathfrak {p}}^{-\beta _\mathfrak {p}}. \end{aligned}$$

If $\chi _{\mathfrak {p}}$ is unramified, then $\beta _{\mathfrak {p}} = 1$, and this factor of $\alpha _{\mathfrak {p}}$ cancels the one appearing in $Q'(\pi _{\mathfrak {p}},\chi _{\mathfrak {p}})$ in Theorem 5.16, leaving exactly $e(\pi _{\mathfrak {p}},\chi _{\mathfrak {p}})$ from Theorem A. If $\chi _{\mathfrak {p}}$ is ramified, then this factor of $\alpha _{\mathfrak {p}}^{-\beta }$ is exactly the difference between $Q'(\pi _{\mathfrak {p}},\chi _{\mathfrak {p}})$ and $e(\pi _{\mathfrak {p}},\chi _{\mathfrak {p}})$.

This gives exactly the interpolation formula of Theorem A, except for one additional term: the sign $\chi _{[p]}(\det w_n)$. To remove this, we simply renormalise $L_p^{i_p}(\tilde{\pi })$ by multiplying it by the Dirac measure $[\det w_n]$, that is, the bounded measure on ${{\,\textrm{Gal}\,}}_p$ defined by $\int _{{{\,\textrm{Gal}\,}}_p} \chi \cdot \textrm{d}[\det w_n] = \chi (\det w_n)$. This process removes the term $\chi _{[p]}(\det w_n)$, proving the claimed interpolation formula and completing the proof of Theorem A. $\square$

### Unicity in the imaginary quadratic case

Now let *F* be imaginary quadratic. The purity condition on $\lambda$ (Definition 2.2) implies that $\lambda _{\sigma ,n} - \lambda _{\sigma ,n+1} = \lambda _{c\sigma ,n} - \lambda _{c\sigma ,n+1} = k$, say, so the range of standard critical infinity types for $\lambda$ forms the $(k+1)\times (k+1)$ square $\{(j_\sigma ,j_{c\sigma }) \in \textbf{Z}^2 : -\lambda _{\sigma ,n+1} \geqslant j_\sigma \geqslant -\lambda _{\sigma ,n}, -\lambda _{c\sigma ,n+1} \geqslant j_{c\sigma } \geqslant -\lambda _{c\sigma ,n}$. The non-*Q*-critical slope bound says, then, that ‘the growth is strictly less than the number of critical values’. Let $\tilde{\pi }$ be as in Theorem A. The following is proved in [35] (cf. [42, §7.5]).

#### Proposition 7.6

If $\tilde{\pi }$ is non-*Q*-critical slope, then $L_p^{i_p}(\tilde{\pi })$ is uniquely determined by its growth and interpolation properties.

When *F* is totally real, the analogous unicity results are discussed in [12, Prop. 6.25]. If *F* is not imaginary quadratic or totally real, unicity remains mysterious; see e.g. [7, §13].

## Data Availability

No new data were created in the preparation of this paper.

## Footnotes

1 Constructions of *p*-adic *L*-functions interpolating other ranges would be extremely interesting; see e.g. [16], which gives an example for ${{\,\textrm{GL}\,}}_2$ over an imaginary quadratic field, assuming $\pi$ is base-change.

2 This is where we want to use $\beta \in \textbf{Z}_{\geqslant 1}^{\mathfrak {p}|p}$, rather than the ‘true’ conductor $p^{\beta _0}$; for *p*-adic interpolation it is necessary to twist non-trivially at each $\mathfrak {p}$, even when the conductor $\beta _{0,\mathfrak {p}}$ itself is trivial.
